# Phage Frontiers: Genomic and Functional Profiling of Novel Virulent Agents Targeting Foodborne *Enterobacteriaceae*

**DOI:** 10.3390/biology15070578

**Published:** 2026-04-04

**Authors:** Ramy Abdelreheim Qabel, Miao Xu, Chunwen Li, Chuhan Zhang, Chuanzhi Zhang, Yong Huang, Guangming Xiong, Edmund Maser, Liquan Guo

**Affiliations:** 1 College of Life Sciences, Jilin Agricultural University, Changchun 130118, China; ramyqabel@azhar.edu.eg (R.A.Q.); miaox@jlau.edu.cn (M.X.);; 2 Agriculture Botany (Microbiology) Department, Faculty of Agriculture, Al-Azhar University, Nasr City, Cairo 11884, Egypt; 3 School of Grain Science and Technology, Jilin Business and Technology College, Changchun 130507, China; 4 Institute of Toxicology and Pharmacology, University Medical School Schleswig-Holstein, 24105 Kiel, Germany

**Keywords:** bacteriophage therapy, Enterobacteriaceae foodborne illness, phage-host interactions, gene prediction algorithms, proteomic analysis

## Abstract

Foodborne pathogens of Enterobacteriaceae are becoming an increasing global concern, with multidrug-resistant strains posing significant risks to food safety and public health, especially in high-risk products like dairy. This research focused on isolating, biologically characterizing, and genomically profiling new bacteriophages that target key Enterobacteriaceae members as potential biocontrol agents. Eight phages were isolated from wastewater using four bacterial hosts and analyzed through transmission electron microscopy, one-step growth analysis, adsorption kinetics, host range evaluation, whole-genome sequencing, comparative genomics, phylogenetic analysis, proteomic profiling, and virion assembly pathway characterization. All eight isolates exhibited icosahedral heads with contractile tails typical of Myoviridae morphology, demonstrated broad-spectrum lytic activity against 21 bacterial strains (infectivity: 47.6–95.2%), showed high adsorption efficiencies (84.75–99.98%), and had burst sizes ranging from 11 to 166 particles per cell. Genome sizes varied from 103 to 170 kb with coding densities between 92–96%. Importantly, none contained antimicrobial resistance genes, virulence factors, or lysogeny-associated elements, confirming their strictly lytic lifestyles and favorable biosafety profiles. Phylogenetic and comparative analyses indicated mosaic genomic structures influenced by horizontal gene transfer rather than host phylogeny. These findings provide a robust biological and genomic basis for evaluating these phages as potentially safe and effective alternatives to antibiotics in controlling foodborne Enterobacteriaceae, pending further in situ validation.

## Simple Summary

Each year, foodborne diseases caused by harmful bacteria impact around 600 million individuals globally, posing a significant risk to public health and food safety. A particularly worrisome group of these bacteria, known as Enterobacteriaceae, has developed increased resistance to antibiotics and the ability to thrive across a wide range of temperatures, complicating the treatment and management of infections. This resistance issue has been exacerbated by the excessive use of antibiotics in agriculture and farming. Dairy products are particularly susceptible to contamination by these bacteria due to their high nutrient content and conditions that favor bacterial growth. This research aimed to identify and describe natural viruses called bacteriophages, which can specifically target and eliminate these harmful bacteria without affecting beneficial microbes or humans. Eight novel bacteriophages were isolated from wastewater and thoroughly assessed for their bacterial-killing ability, genetic composition, and safety. All eight demonstrated a strong capacity to eradicate a broad spectrum of harmful bacterial strains, including those resistant to antibiotics. Genetic analysis confirmed that none of them carry harmful genes, making them safe candidates for food use. These results indicate that bacteriophages could be effective, natural, and safe alternatives to antibiotics for safeguarding food products from bacterial contamination, supporting global efforts to combat antibiotic resistance.

## 1. Introduction

The global food industry is currently confronting a rising crisis related to foodborne pathogens that annually harm around 600 million individuals. The Enterobacteriaceae (multidrug-resistant; MDR) exhibit high antimicrobial characteristics [1,2]. This crisis is aggravated by the extensive use of antibiotics in animal and agricultural sectors [3]. Dairy products represent high-risk food matrices particularly susceptible to microbial contamination and pathogen proliferation due to their intrinsic physicochemical properties and processing characteristics. The rich nutritional composition of dairy products, including high protein content, lactose, and essential vitamins and minerals, provides an ideal substrate for microbial growth. Furthermore, the near-neutral pH (6.8–7.2) of most dairy products, combined with high water activity (aw > 0.95) and optimal storage temperatures, creates favorable conditions for the survival and multiplication of various foodborne pathogens including *Salmonella* spp., *Listeria monocytogenes*, *Escherichia coli*, and *Staphylococcus aureus* [4,5,6,7,8].

Bacteriophages represent promising alternatives to traditional antimicrobials due to their high specificity for target bacteria, self-limiting replication, minimal impact on beneficial microflora, and evolutionary adaptability, which potentially avoids resistance development [9,10]. Effective phage-based biocontrol requires comprehensive characterization encompassing host range, lytic efficiency, replication kinetics, and genomic analysis for lifestyle determination, safety assessment, and evolutionary relationships [11,12]. Despite the growing application of next-generation sequencing to phage genomics, critical knowledge gaps persist: the genomic diversity of phages specifically targeting foodborne Enterobacteriaceae remains poorly characterized; the correlations between genomic architecture and biological properties such as host range and burst size are insufficiently explored; and the criteria defining optimal therapeutic phages for food safety applications have not been systematically established [13,14]. Furthermore, no bacteriophage targeting *Obesumbacterium proteus*, a recognized Enterobacteriaceae contaminant in dairy and brewery environments, has been described to date, representing a significant gap in phage-based biocontrol resources.

This study aims to characterize the comprehensive biological, genomic, and proteomic aspects of novel bacteriophages targeting a clinically and industrially significant member of the Enterobacteriaceae. The efficacy of these bacteriophages as biological control agents will be systematically evaluated to determine their potential application in mitigating foodborne pathogens and enhancing food safety strategies. Significantly, as far as we are aware, Phage_Ob_P is the first bacteriophage identified to target *Obesumbacterium proteus*. This bacterium, mainly recognized as a contaminant within the Enterobacteriaceae family, has not had any associated phages documented in scientific literature or included in publicly accessible genomic databases until now.

## 2. Materials and Methods

### 2.1. Isolation and Identification of Bacterial Strains and Prophage Induction

Fifteen raw milk samples were collected from dairy cows in Changchun City, Jilin Province (located at the Northern East Part of China), using sterile protocols, transported via cold-chain, and processed within 24 h following standard microbiological techniques [15,16], with TSB (tryptic soy broth) enrichment at 37 °C, 180 rpm for 18–24 h followed by selective cultivation on TSIA (triple-sugar iron agar) and systematic colony purification through repeated streaking to obtain pure cultures. Bacterial identification employed direct amplification of the 16S rRNA gene using standardized PCR with 35 thermal cycles (94 °C denaturation, 54 °C annealing, 72 °C extension) using universal 16S rRNA primers 27F (5′-AGAGTTTGATCMTGGCTCAG-3′) and 1492R (5′-TACGGYTACCTTGTTACGACTT-3′) [15], agarose gel electrophoresis for product verification, and sequencing with comparative NCBI database analysis for taxonomic classification. Prophage induction employed Mitomycin C at 0.5 μg/mL and 10 μg/mL on bacterial cultures grown to late logarithmic phase (OD600 = 0.4), followed by extended incubation, centrifugation and filtration to concentrate potential phage particles, and spot assay testing [17], to confirm prophage induction and lytic activity.

### 2.2. Bacteriophage Isolation, Purification and Preparation of Phage Stock

Specimens potentially containing bacteriophages were collected from a sewage treatment facility in Changchun City, Jilin Province, China. Bacterial host strain cultures were prepared in autoclaved tryptic soy broth (TSB), into which 5 mL of effluent and sewage samples were inoculated alongside 1 mL of overnight bacterial culture in 50 mL TSB, then incubated at 37 °C for 24 h at 180 rpm. The phage lysate was extracted by centrifugation at 10,000 rpm for 20 min at 4 °C, followed by treatment with 10% chloroform, vigorous shaking, and re-centrifugation under identical parameters. Supernatants were subsequently filtered through 0.45 μm Millipore filters, collected into sterile tubes, and stored at 4 °C, following the protocol established by [18]. Phage detection utilized spot test protocols with soft agar overlay techniques for rapid preliminary screening [19], while quantitative plaque assays employed serial dilution with double-layer agar systems and standardized incubation (37 °C, 16–24 h) for enumeration with provisions for plaque isolation and transfer into CM buffer (2.5 g/L MgSO_4_·7H_2_O; 0.735 g/L CaCl_2_; 0.05 g/L gelatin; 6 mL/L 1 M Tris, pH 7.5).

To obtain highly virulent phages, individual plaques exhibiting varying lysis degrees were isolated using a sterile cork borer (Sigma-Aldrich, St. Louis, MO, USA) in 500 μL of CM buffer and maintained at room temperature overnight. Plaque assays were performed at least three times per isolate until uniform plaque morphology was achieved. For lysate production, purified plaques were enriched in 3 mL TSB containing fresh bacterial culture and incubated overnight at 37 °C with agitation at 180 rpm. For high-quality lysate preparation, individual plaques were resuspended in 0.5 mL CM buffer, combined with 1 mL of fresh bacterial culture (109 CFU/mL) in 25 mL TSB, and incubated for 24 h at 37 °C with agitation. In both procedures, suspensions were centrifuged at 10,000 rpm for 20 min at 4 °C, supernatants treated with 10% chloroform, agitated for 5 min, equilibrated at room temperature for 30 min, and re-centrifuged. Final supernatants were filtered through 0.45 μm Millipore filters and stored at 4 °C for future use, as described by [19,20].

Bacteriophages were concentrated and purified using a dextran sulfate-polyethylene glycol two-phase liquid system following the methodology of [21]. This method was selected over ultracentrifugation because it is scalable to large volumes, avoids mechanical shearing of phage particles, and has been shown to preserve phage infectivity while effectively removing host cell debris and bacterial proteins [21]. Briefly, 160 gm of PEG, 4 gm of dextran sulfate, and 116.88 g of NaCl were added to 2 L of phage lysate, vigorously agitated, and stored at 4 °C for 24 h. Following phase separation, the turbid bottom layer was centrifuged at 3000 rpm for 10 min, and the resulting interface was resuspended in 20 mL of borate buffer. Dextran sulfate was precipitated by adding 2.0 mL of 3 M potassium chloride, refrigerated at 4 °C for 2 h, and removed by centrifugation at 3000 rpm for 10 min. The lysate was then dialyzed in a dialysis bag against 5 L of (ddH_2_O) with magnetic stirring at 4 °C for 24 h. Supernatants were filtered through 0.45 μm Millipore filters, and phage particles were pelleted by centrifugation at 15,000 rpm. Pellets were resuspended in CM buffer and stored at 4 °C for subsequent studies.

### 2.3. Bacteriophage Morphological and Physiological Characterization

Transmission electron microscopy employed high-speed centrifugation (15,000 rpm) of concentrated phage lysates (10^9^ particles/mL), washing with CM buffer, application to carbon-coated Formvar grids, negative staining with 2% phosphotungstic acid at pH 7.2, and examination using HITACHI-HT7800 instrumentation (Hitachi High-Tech Corporation, Tokyo, Japan) [22], for structural characterization and taxonomic classification.

One-step growth experiments were performed to determine the burst size and latent period of selected phages, following the methodology of [23]. Phages were mixed with bacterial hosts at a multiplicity of infection (MOI) of 0.1 and incubated at 37 °C with agitation at 180 rpm for 10 min. At 10.5 min post-infection, 100 μL were transferred into 9.9 mL of fresh TSB and serially diluted twice into 9 mL TSB volumes with gentle agitation. All three tubes were incubated at 37 °C at 180 rpm for 10 min. Beginning at 11 min post-infection, 100 μL aliquots were plated at 10-min intervals over 240 min by combining with 1000 μL of indicator bacteria and 4 mL of overlay medium onto TSA plates. Plaques were enumerated after 24 h incubation at 37 °C. The relative burst size was calculated as follows: Relative burst size = (Final titer − Initial titer)/Initial titer.

Phage adsorption kinetics were evaluated by combining phage isolates with bacterial cultures (OD_600_ = 0.4–0.6) in CM buffer at an MOI of 10, incubated at 37 °C with agitation at 180 rpm [24]. At predetermined intervals (0, 10, 20, 30, 40, 50, and 60 min), aliquots were withdrawn and centrifuged at 6000 rpm for 20 min at 4 °C to pellet phage-bacterial complexes. Supernatants were filtered through 0.45 μm Millipore filters, and unadsorbed phage concentrations (Pₜ) were quantified by standard double-layer plaque assay following 24 h incubation at 37 °C. Adsorption parameters were calculated using the following equations:

Adsorption Capacity = P_0_ − P_t_, Adsorption Rate Constant (K) = (P_0_ − Pₜ)/(P_0_ × B × t), Adsorption Velocity (ν) = K × B, Adsorption Efficiency (%) = [(P_0_ − P_60_)/P_0_] × 100

where P0 is the initial phage titer, P60 the unadsorbed phage concentration at 60 min, B the bacterial concentration (CFU/mL), and t the time in minutes [25,26]. Linear regression analysis with Pearson correlation coefficients (R^2^) was performed on K versus ν data to validate first-order binding behavior and experimental reproducibility. Normalised adsorption progress curves were generated by plotting [(P_0_ − Pₜ)/P_0_ × 100] against time to enable comparative kinetic analysis across phage isolates and host strains independent of absolute phage titers [27].

To assess phage host specificity, ten water samples from various lakes and eight animal waste samples (chicken, goat, cow, duck, and turkey manure) were collected from livestock farms in Changchun City, Jilin Province, China. Microbial isolation, purification, and identification were conducted as previously described [28]. Host range was initially evaluated by spotting 10 μL of serial phage dilutions (100 to 10^8^) onto TS soft agar (0.4%) incorporated with individual bacterial strains, followed by overnight incubation at 37 °C. Plates were examined for isolated plaques indicating completion of a full lytic cycle. Phages exhibiting the broadest bactericidal range in spot tests were subsequently selected for comprehensive evaluation of productive infection through efficiency of plating (EOP). Each phage was tested in triplicate at four dilutions against all susceptible strains using stationary-phase bacteria cultivated overnight (18 h) at 37 °C, with 1000 μL of each culture combined with 100 μL of diluted phage lysate in double-layer plaque assays. EOP values were classified as follows: high (≥0.5), medium (0.1–0.5), poor (0.001–0.1), and inefficient (≤0.001), as described by [29].

### 2.4. Bacteriophage Genome Analysis

High-quality phage genomic DNA was extracted following [17], with modifications. The protocol involved DNase I and RNase A treatment, overnight incubation, proteinase K digestion with SDS and EDTA at 65 °C, phenol: chloroform: isoamyl alcohol purification, and ethanol precipitation. DNA concentration was determined using Nanodrop spectrophotometry. Eight bacteriophage isolates were sequenced using Whole Genome Shotgun methodology by Shanghai Personalbio Technology Co., Ltd. (Shanghai, China). De novo assembly utilized A5-MiSeq [30] and SPAdes [31], for de novo contig construction following adapter sequence removal, with depth-based sequence extraction and NCBI blastn comparison [32]. Assembly refinement employed MUMmer colinearity analysis [33] for gap filling and Pilon corrections [34], to generate final genome sequences.

Viral genome quality was assessed using CheckV [35], through automated analysis of single-contig sequences. The pipeline identified non-viral regions, classified genes as viral or microbial, estimated completeness against viral genome databases, and assigned five-tier quality classifications: Complete, High quality (>90%), Medium quality (50–90%), Low quality (0–50%), and Undetermined. Phage lifestyles were predicted using BACPHLIP [36], employing HMMER3 to identify lysogeny-associated protein domains and Random Forest classifiers for lifestyle determination. The tool provided temperate or virulent classifications with probability scores for confidence assessment.

Genome annotation was performed using Pharokka [37], identifying ORFs, CDS, tRNAs, tmRNAs, and CRISPR arrays. Functional annotation utilized the PHROGs database, generating GenBank-compliant output with gene coordinates, product names, and functional assignments.

Annotation comparative was evaluated across seven tools: Pharokka [37], Prokka [38], Phanotate [39], GeneMark [40], Glimmer [41], Decouphage [42], and Prodigal [43]. Multiple tools were employed because no single gene prediction algorithm performs optimally across all phage genome types, particularly given the prevalence of non-canonical start codons, overlapping genes, and unique codon usage biases in bacteriophage genomes. The consensus strategy adopted here defined a gene as high-confidence when predicted by four or more of the seven tools, thereby minimizing false positives while retaining sensitivity. Pharokka annotations, which integrate the PHROGs functional database, were used as the primary reference for downstream functional analysis. Quantitative agreement was assessed using UpSet plots [44], with consensus genes predicted by four or more tools. Jaccard indices were employed to evaluate tool concordance.

Comparative genomic analysis was conducted using CompareM v0.1.0 [45] to calculate pairwise amino acid identity (AAI) values between eight bacteriophage isolates using the aai_wf command with default parameters. Gene prediction was performed using Prodigal [43], followed by protein sequence alignments using DIAMOND v0.9.0 with an e-value threshold of 1 × 10^−5^, minimum sequence identity of 30%, and minimum alignment length of 70% [46].

Phylogenetic analysis utilized terminase large subunit sequences retrieved via BLAST (version 2.14), aligned with MUSCLE in MEGA12 [47], and analyzed using maximum likelihood methods with 1000 bootstrap replicates [48,49]. Trees were visualized using iTOL v6 [50].

### 2.5. Bacteriophages Proteomic Analysis

Virion proteins were identified using DeePVP [51], employing convolutional neural networks for PVP prediction (threshold 0.5) and classification into ten functional classes including major capsid, tail, and baseplate proteins. PVP distribution analysis identified functionally significant regions across genomes.

Bacteriophage structural proteins were validated through SDS-PAGE following [52], with modifications by [53]. Phage particles were purified using polyethylene glycol precipitation and cesium chloride density gradient ultracentrifugation. Proteins were denatured in Laemmli buffer containing 2% SDS and 5% β-mercaptoethanol, then separated on discontinuous gels (4% stacking, 12% resolving polyacrylamide) at 120 V and 150 V respectively. Molecular weights were estimated using Precision Plus Protein Dual Color Standards, followed by Coomassie Brilliant Blue R-250 staining. Digital analysis was performed using ImageLab software (version 6.2, Bio-Red) with three technical replicates per isolate for computational prediction validation.

The Kegg Functional pathway analysis was performed using PHANOTATE v1.5.0 [39], with mapping to the KEGG database facilitated by the BlastKOALA/GhostKOALA servers [54,55].

## 3. Results

### 3.1. Bacterial Isolation, Identification, and Prophage Induction

This paper isolated, identified, and characterized Enterobacteriaceae from raw milk were isolated, identified, and characterized, establishing a connection between contamination and various environmental factors, including cowshed conditions, bedding, seasonal feed, teat hygiene, and equipment cleanliness. Taxonomic analysis was performed for five species encompassing *E. coli* str. K-12 (40%, n = 6), *S. boydii* strain ESBL-W3-2 (33%, n = 5), *R. ornithinolytica* strain YZSHE173 (13.3%, n = 2), and *O. proteus* strain LE8 and S. sonnei strain SE6-1 (6.66% each, n = 1; Figure S1). To complement the bacterial characterization, prophage induction experiments were conducted using Mitomycin C at concentrations of 0.5 μg/mL and 10 μg/mL during the logarithmic growth phase. Despite observable increases in culture turbidity across all tested strains, no detectable plaque formation occurred under either treatment condition. These findings indicated absence of inducible temperate phages within the isolated bacterial populations, suggesting that these Enterobacteriaceae strains had no lysogenic bacteriophages under the experimental conditions employed.

### 3.2. Isolation, Purification and TEM Investigations of Specific Bacteriophages

**Figure 1:**
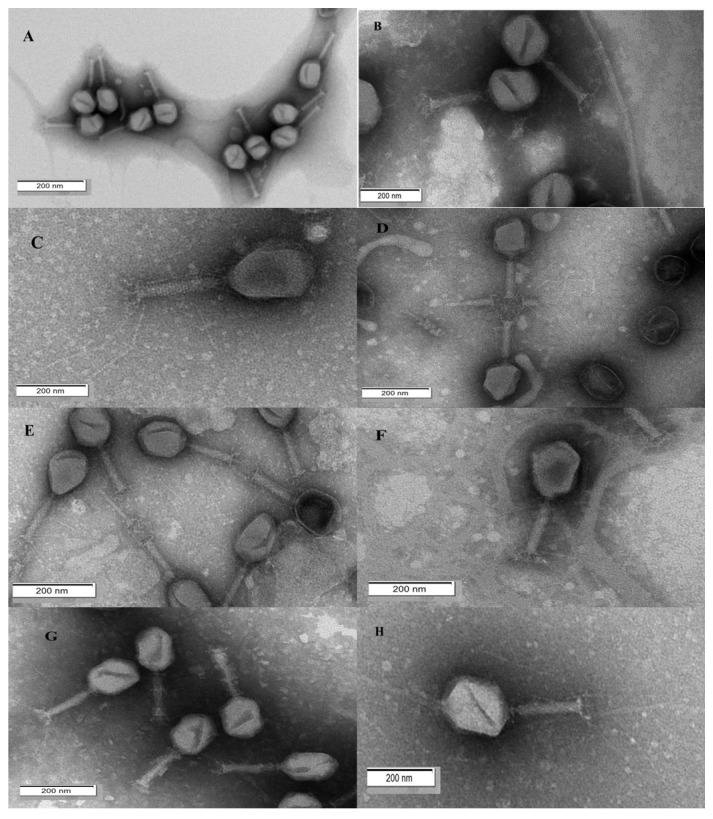
Transmission Electron Microscopy of Bacteriophage Isolates. This figure illustrates negatively stained transmission electron micrographs that reveal the distinct morphological features of eight bacteriophage isolates derived from environmental samples. (**A**–**H**) depict Phage OES_C-1, Phage OES_C-2, Phage OES_C-3, Phage_SH-1, Phage_SH-2, Phage_Ra_O-1, Phage_Ra_O-2, and Phage_Ob_P, respectively. All isolates exhibit morphology consistent with the Myoviridae family, characterized by icosahedral heads containing the viral genome and contractile tail structures essential for host cell infection.

Bacteriophages were isolated from wastewater and sewage samples using four bacterial hosts: *E. coli*, *S. boydii*, *R. ornithinolytica*, and *O. proteus*. Top-agar spot assays with 20-μL samples showed large, clear lytic zones, indicating high concentrations of bacteriophage activity against all strains (Figure S2). Plaque assays confirmed the presence of viable phages through distinct plaque formations, which varied in appearance, with some having prominent halos and others appearing as small pinpoint without halos (Figure S3, Table S2). Eight bacteriophages were purified through multiple rounds of single-plaque selection and serial dilutions (1:10) in CM buffer: three targeting *E. coli* (Phage OES_C-1, Phage OES_C-2, Phage OES_C-3), two targeting *S. boydii* (Phage_SH-1, Phage_SH-2), two targeting *R. ornithinolytica* (Phage_Ra_O-1, Phage_Ra_O-2), and one targeting *O. proteus* (Phage_Ob_P; Table S1). Each isolated bacteriophage was assigned a unique accession number as shown in Table S1. Transmission electron microscopy, following differential centrifugation at 15,000 rpm and phosphotungstic acid staining, revealed that all isolates had icosahedral heads and long contractile tails typical of Myoviridae (Figure 1). Head diameters ranged from 99 nm for Phage OES_C-1 to 118 nm for Phage_Ra_O-1, with tail lengths between 95–125 nm. Phage_Ra_O-1 had the largest dimensions (118 nm head, 114 nm tail), while the other isolates had head diameters between 101–107 nm and tail lengths between 95–107 nm (Table S2).

### 3.3. Bacteriophage Growth Dynamics and Lytic Parameters

**Figure 2:**
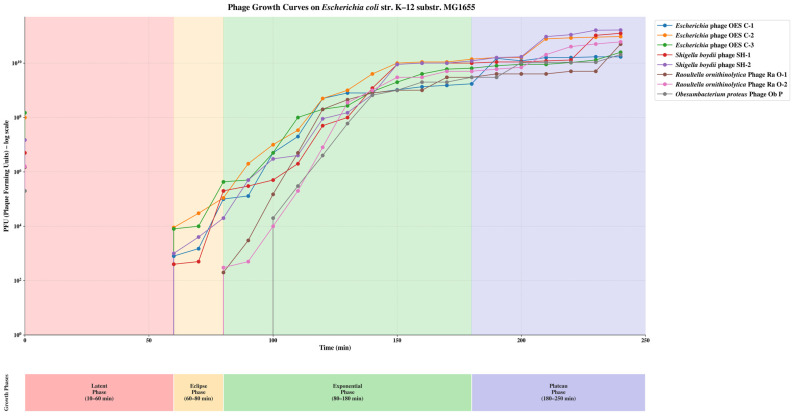
One-step growth curves of bacteriophage isolates representing distinct morphotypes, analyzed using *E. coli* as the host organism. The production analysis identified three distinct categories of phage performance. The phages with the highest production were Phage_SH-2 (1.65 × 10^11^ PFU, burst size: 11 particles/cell) and Phage_SH-1 (1.25 × 10^11^ PFU, burst size: 25 particles/cell), which demonstrated exceptional cross-genus infectivity despite their relatively small burst sizes. In contrast, phages with high burst sizes included Phage OES_C-3 (2.5 × 10^10^ PFU, burst size: 166 particles/cell), Phage OES_C-1 (1.7 × 10^10^ PFU, burst size: 112 particles/cell), and Phage_Ob_P (2.05 × 10^10^ PFU, burst size: 103 particles/cell), indicating efficient replication per infection cycle. A third category, comprising balanced producers, included Phage OES_C-2 (9.50 × 10^10^ PFU, burst size: 95 particles/cell), Phage_Ra_O-2 (6.05 × 10^10^ PFU, burst size: 38 particles/cell), and Phage_Ra_O-1 (5 × 10^10^ PFU, burst size: 33 particles/cell), which exhibited moderate burst sizes with high total yields.

One-step growth experiments were conducted for eight bacteriophage isolates across four Enterobacteriaceae hosts, revealing intricate and sometimes unexpected cross-species infectivity patterns. On *E. coli*, all isolates displayed a typical latent phase (0–60 min), an eclipse phase (60–80 min), and an exponential phase (80–180 min). Phage_SH-1 and Phage_SH-2 reached the highest final titers (1.25 × 10^11^ and 1.65 × 10^11^ PFU at 240 min), despite originating from a different host. Burst sizes varied from 11 to 166 particles/cell, with native *Escherichia* phages generally showing higher values (95–166 particles/cell), indicating optimal host-phage co-adaptation (Figure 2). On *S. boydii*, native Phage_SH-1 and Phage_SH-2 showed superior replication efficiency with shorter latent periods (30–40 min) and higher final titers (6.05 × 10^10^ and 1.50 × 10^11^ PFU), while heterologous phages had longer latent phases (60–90 min) and lower titers, although burst sizes were relatively consistent across isolates (66–110 particles/cell) (Figure S4). On *R. ornithinolytica*, an unexpected reversal of host permissiveness was noted, with heterologous Phage_OES_C-1 achieving the highest final titer (7 × 10^10^ PFU), significantly exceeding native phages Phage_Ra_O-1 and Phage_Ra_O-2 (2 × 10^9^ and 2.50 × 10^9^ PFU), suggesting strain-specific resistance mechanisms or receptor modifications that limit homologous phage infection (Figure S5). On *O. proteus*, heterologous phages also outperformed the native Phage_Ob_P (6 × 10^9^ PFU), with Phage_Ra_O-2 reaching the highest titer (1.50 × 10^10^ PFU) and burst sizes ranging from 25 to 133 particles/cell (Figure S6). Biphasic exponential kinetics observed in several isolates suggest complex population dynamics, potentially involving phage-induced physiological changes in the host. The replication kinetics data collectively demonstrated that the bacteriophages characterized in this study exhibited broad and adaptable infectivity across multiple Enterobacteriaceae hosts, surpassing conventional host-specificity paradigms. The superior performance of heterologous phages on *R. ornithinolytica* and *O. proteus* indicates that phylogenetic proximity alone does not govern replication success; rather, receptor compatibility, host defense mechanisms, and intracellular replication dynamics collectively determine phage-host interaction outcomes. Variability in latent periods, burst sizes, and final titers across host-phage combinations further underscores the adaptive plasticity of these phages, a highly desirable attribute for biocontrol applications.

### 3.4. Bacteriophage Host Adsorption Kinetics and Binding Efficiency

**Figure 3:**
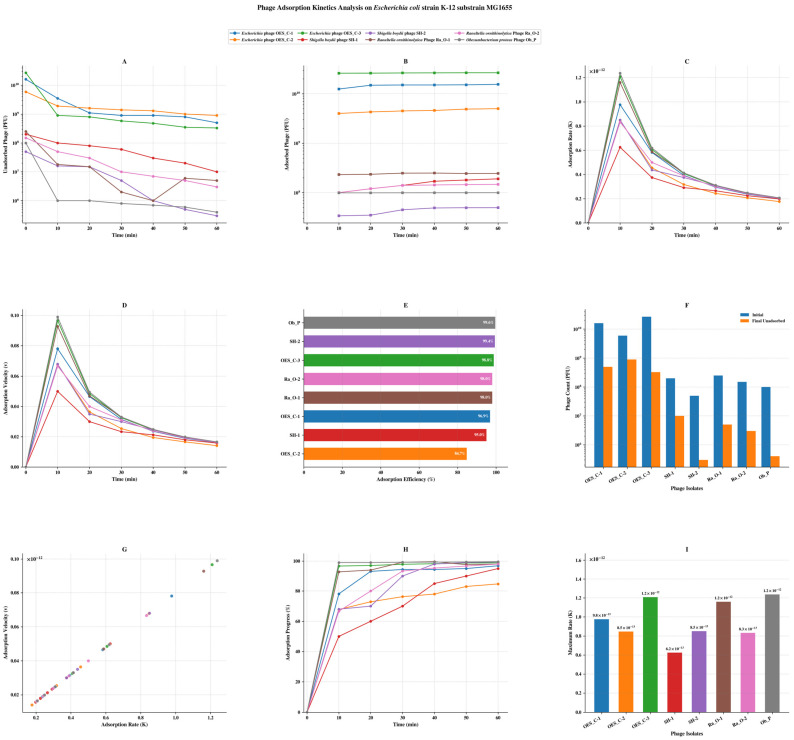
illustrates the adsorption kinetics of eight bacteriophage isolates (Phage OES_C-1, Phage OES_C-2, Phage OES_C-3, Phage_SH-1, Phage_SH-2, Phage_Ra_O-1, Phage_Ra_O-2, and Phage_Ob_P) on *E. coli*. The temporal profiles depict. (**A**) the decline of un-adsorbed phages, (**B**) the accumulation of adsorbed phages, (**C**) the adsorption rate (K), (**D**) the adsorption velocity (ν), (**E**) the final adsorption efficiency, (**F**) a comparison of initial versus final un-adsorbed phages, (**G**) the correlation between rate and velocity, (**H**) the normalized adsorption progress, and (**I**) a comparison of maximum adsorption rates. All isolates demonstrated rapid biphasic attachment kinetics, achieving binding efficiencies exceeding 8e5% within the 60-min observation period. Peak adsorption rates were recorded at the 10-min mark, ranging from 6.25 × 10^−13^ to 1.24 × 10^−12,^ after which binding rates gradually decreased, consistent with pseudo-first-order kinetics and surface receptor depletion. Among the tested phages, Phage_Ob_P and Phage OES_C-3 exhibited superior binding performance, with efficiencies surpassing 99% and achieving over 90% attachment within the initial 10 min. The strong linear relationships between adsorption rate and velocity parameters (R^2^ > 0.95) across all phages confirm experimental reliability and support first-order binding kinetics.

The adsorption kinetics of eight bacteriophage isolates were examined across four Enterobacteriaceae host strains, revealing highly efficient binding profiles and significant receptor promiscuity. Against *E. coli*, all isolates demonstrated classical biphasic adsorption curves with high efficiencies, with Phage_Ob_P achieving the highest cross-species adsorption (99.60%; maximum rate: 1.24 × 10^−12^ at 10 min) despite originating from a phylogenetically distinct host. Native *Escherichia* phages exhibited robust homologous binding (84.75–98.77%), while heterologous *Shigella* and *Raoultella* phages achieved comparably high efficiencies (95–99.40%), suggesting conserved lipopolysaccharide or outer membrane protein receptor structures across Enterobacteriaceae genera (Figure 3). Against *S. boydii*, homologous phages Phage_SH-1 and Phage_SH-2 demonstrated near-complete receptor saturation within 10 min (99.93% and 99.98%, respectively; maximum rates: 1.87–1.88 × 10^−12^), consistent with highly optimised coevolved tail fiber-receptor interactions. Heterologous *Raoultella* phages also exhibited strong cross-genus binding (90–99.67%), reflecting conserved surface epitopes between *Shigella* and *Raoultella* genera (Figure S7). Against *R. ornithinolytica*, native Phage_Ra_O-1 and Phage_Ra_O-2 achieved the highest efficiencies (99.94% and 99.82%; maximum rates: 9.89 × 10^−12^ and 8.55 × 10^−12^), while non-host phages similarly demonstrated strong cross-species adsorption (90–99.64%), suggesting structural receptor similarities across host genera (Figure S8). Against *O. proteus*, heterologous phages Phage_OES_C-1 and Phage_OES_C-3 achieved the highest adsorption efficiencies (99% and 99.50%), marginally exceeding the native Phage_Ob_P (98%), with 70–87.5% of total binding occurring within the first 10 min across all isolates, indicative of receptor saturation, adsorption–desorption equilibrium, and potential steric hindrance effects at high surface occupancy. First-order kinetic behaviour was confirmed across all host systems by strong positive linear correlations between adsorption rate and velocity parameters (R^2^ > 0.92–0.95) (Figure S9). The adsorption kinetics data collectively revealed that the bacteriophages examined in this study exhibited remarkable binding adaptability across a wide range of phylogenetically diverse Enterobacteriaceae hosts, consistently achieving high adsorption efficiencies regardless of the host’s origin. The nearly universal achievement of over 90% adsorption efficiency in various host-phage pairings strongly suggests that the conservation of crucial surface receptor structures, particularly lipopolysaccharide components and outer membrane proteins, plays a significant role in facilitating broad-spectrum phage attachment within the Enterobacteriaceae family. The rapid initial binding kinetics, with most adsorption occurring within the first 10 min across all host systems, further highlight the efficient receptor recognition capabilities of these phages. From a biocontrol perspective, these high adsorption efficiencies (84.75–99.98%) are biologically significant because they indicate that the phages can rapidly and effectively bind to target bacteria even at relatively low phage-to-bacterium ratios, a critical parameter for practical food safety applications where high phage titers may be difficult to maintain. The burst sizes observed (11–166 particles/cell) span a biologically meaningful range: phages with smaller burst sizes (Phage_SH-1: 11; Phage_SH-2: 25) compensate through exceptionally high total titers, suggesting rapid cell lysis cycles rather than accumulation of progeny, while phages with larger burst sizes (Phage_OES_C-3: 166; Phage_OES_C-1: 112) are better suited for applications requiring exponential amplification from low initial concentrations. Together, these kinetic parameters support the suitability of these phages for inclusion in multi-phage biocontrol cocktails.

### 3.5. Bacteriophage Host Range and Plating Efficiency Analysis

The infectivity spectrum and plating efficiency of eight bacteriophage isolates were systematically assessed against 21 bacterial strains, revealing diverse host specificity patterns across Enterobacteriaceae species (Figure S10). Phage_SH-2 demonstrated the broadest host range, infecting 95.2% of tested strains, followed by Phage_OES_C-1 (76.1%) and Phage_Ra_O-2 (71.5%). Phage_OES_C-2, Phage_SH-1, Phage_OES_C-3, and Phage_Ra_O-1 exhibited intermediate infection rates (57.1–61.9%), while Phage_Ob_P showed the most restricted range (47.6%).

Host-specific infection patterns were observed across all four primary host strains. *E. coli* displayed the most permissive phenotype, supporting productive infection by all eight phages (EOP: 1.0–25.0), consistent with its well-characterised surface receptor accessibility. *S. boydii* showed broad but variable susceptibility, with homologous Phage_SH-1 and Phage_SH-2 achieving high efficiency (EOP: 1.0), while heterologous phages yielded reduced but appreciable EOPs (0.08–1.93), reflecting the close phylogenetic relationship and shared receptor architectures between *Escherichia* and *Shigella*. Notably, *S. flexneri* KS7 demonstrated selective high-efficiency infection by *Escherichia* phages (EOP: 1.2–3.16) over congeneric *Shigella* phages (EOP: 0.08–0.2), suggesting strain-specific receptor polymorphisms. *R. ornithinolytica* YZSHE173 exhibited a restricted host range, supporting high-efficiency infection exclusively by homologous Phage_Ra_O-1 (EOP: 4.12) and moderate sensitivity to Phage_OES_C-1 (EOP: 0.31), indicating genus-specific receptor requirements. *O. proteus* LE8 displayed intermediate susceptibility (EOP: 0.0–3.0), with Phage_OES_C-1 achieving the highest efficiency (EOP: 0.35). Most clinical *E. coli* strains exhibited highly restricted susceptibility (EOP: 0.0–0.2), highlighting the significant role of strain-level variation in surface receptor expression, capsule composition, restriction-modification systems, and prophage-mediated superinfection exclusion. *C. sakazakii* and *K. variicola* demonstrated narrow host ranges with selective susceptibility to Phage_Ra_O-2 (EOP: 0.32 and 4.0, respectively), suggesting cross-genus tail fiber recognition capability. *S. sonnei* and most *S. flexneri* strains showed broad resistance (EOP: 0.0–0.06), except *S. flexneri* MCURPq_BL04, which exhibited high susceptibility to Phage_Ra_O-2 (EOP: 2.4).

The host range and plating efficiency data collectively highlight the significant diversity in phage-host interactions within Enterobacteriaceae, influenced by a complex mix of receptor polymorphisms, surface structure changes, restriction-modification systems, and internal defense mechanisms. Although E. coli consistently acted as a broadly permissive universal host, the notable variation in EOP values among clinical and environmental strains indicates that species-level susceptibility is not a reliable predictor of strain-level infectivity. The wide host range of Phage_SH-2, Phage_OES_C-1, and Phage_Ra_O-2, along with the cross-genus recognition abilities shown by several isolates, marks these phages as particularly promising for broad-spectrum biocontrol applications.

### 3.6. Comprehensive Genomic Analysis of Bacteriophage Isolates

High-throughput whole-genome sequencing of eight bacteriophage isolates yielded high-quality datasets, with read counts ranging from 5,140,804 (Phage_SH-1) to 9,229,620 (Phage_SH-2). The GC content varied between 35.21% (Phage OES_C-3) and 40.07% (Phage_Ob_P), with a predominance of adenine and thymine (A: 29.5–31.88%; T: 29.82–32.94%), consistent with lower GC content. Notably, Phage_Ra_O-1 and Phage_Ra_O-2 exhibited elevated GC percentages (39.5–39.52%) and increased guanine proportions (18.91–20.62%), suggesting host-specific evolutionary adaptations. Genome assembly revealed substantial architectural diversity: seven phages displayed circular topology, while Phage_SH-2 exhibited a linear configuration, with genome sizes ranging from 103,231 bp (Phage_Ob_P) to 170,469 bp (Phage OES_C-2). CheckV quality assessment confirmed high-confidence status for all genomes, with phages (Phage OES_C-1, Phage OES_C-2, and Phage OES_C-3) showing proviral lengths of 257–264 bp, perfect host gene matches (100%), and maximum completeness scores (1.0). Phage_SH-1 and Phage_SH-2 exhibited 252–260 bp proviral lengths with 100% host gene content and completeness of 1.0, while Phage_Ra_O-1 and Phage_Ra_O-2 displayed consistent 239 bp proviral lengths with high host gene matches (99.81% and 99.41%) and maximum completeness. Phage_Ob_P showed the shortest proviral length (121 bp) and lowest host gene match (91.34%) while maintaining high-confidence classification and maximum completeness. BACPHLIP lifestyle prediction revealed predominantly virulent characteristics across all isolates, with Phage OES_C-1 and Phage OES_C-2 demonstrating strict virulent classification (virulent score = 1.0; temperate = 0), while the remaining phages exhibited high virulence probabilities (0.975–0.9875) with minimal temperate potential (0.0125–0.025), suggesting favorable suitability for therapeutic applications, pending experimental validation.

**Figure 4:**
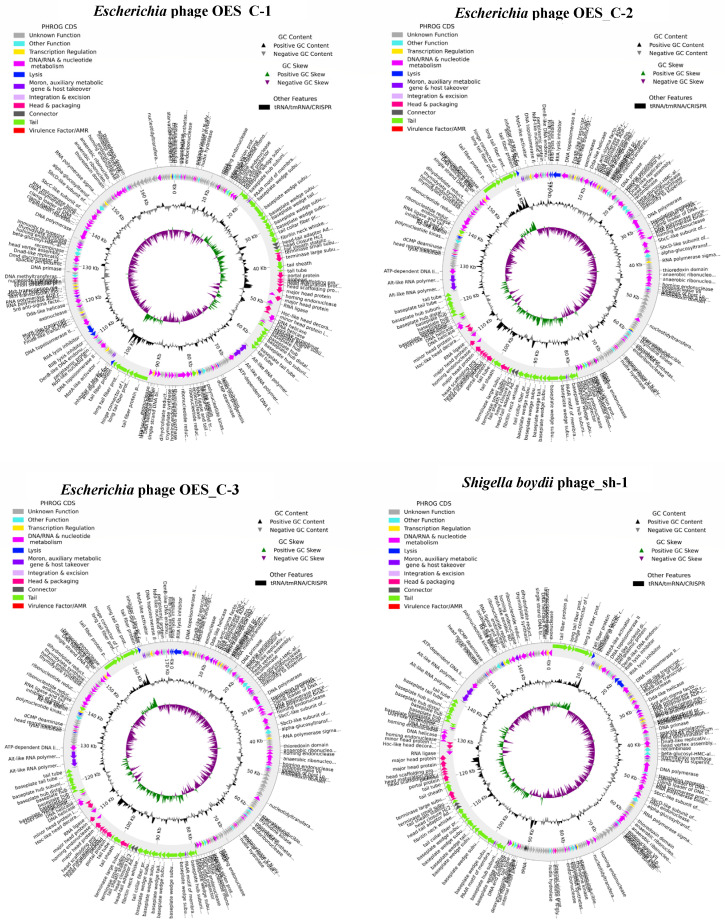

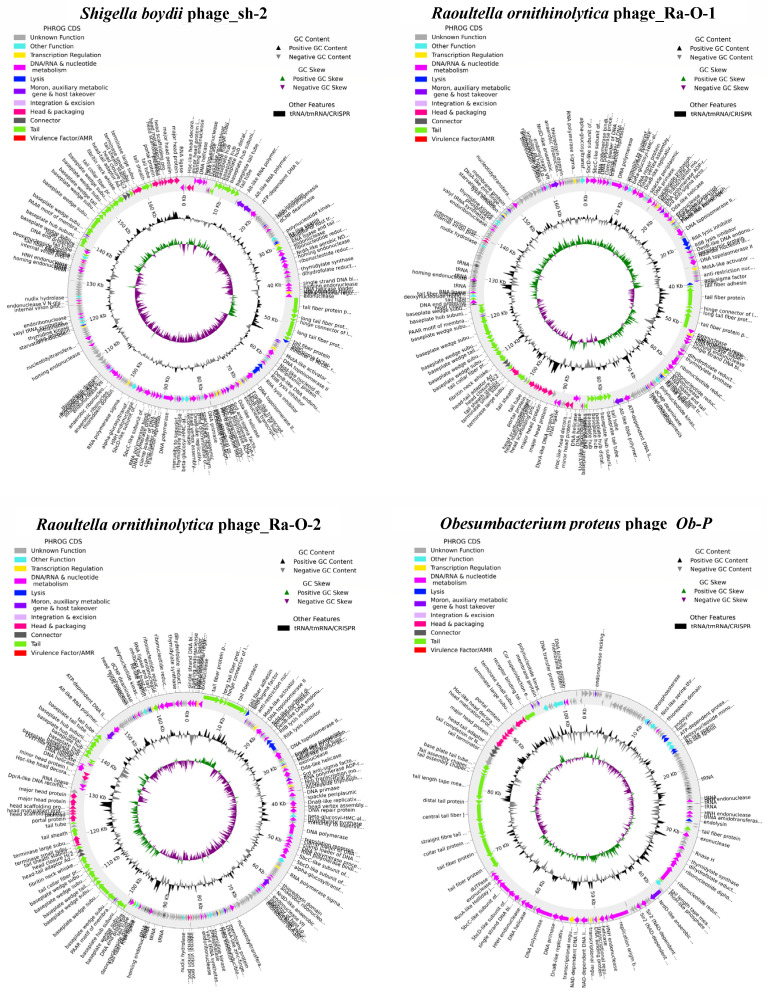
Circular genome maps and functional annotation of Phage OES_C-1, Phage OES_C-2, Phage OES_C-3, Phage_SH-1, Phage_SH-2, Phage_Ra_O-1, Phage_Ra_O-2 and Phage_Ob_P. Each circular representation delineates the comprehensive genomic organization and functional annotation of the phage genomes, as classified by PHROG (Prokaryotic Virus Remote Homologous Groups). The visualization employs a color-coded system to categorize genes by function: unknown function (gray), other function (cyan), transcription regulation (yellow), DNA/RNA & nucleotide metabolism (pink), lysis (dark blue), moron/auxiliary metabolic genes & host takeover (purple), integration & excision (light blue), head & packaging (orange), connector (light green), tail (bright green), and virulence factors/AMR (red). The outermost layer displays gene annotations radiating from the circular genome, indicating specific gene products and their genomic positions. The inner concentric rings exhibit GC content variations (black histogram showing positive and negative GC content) and GC skew (positive and negative strands), which are crucial for identifying replication origins and termini. Additional genomic features such as tRNA, tmRNA, and CRISPR elements are annotated when present.

The genome annotation conducted using Pharokka revealed a diverse array of genetic architectures with consistent functional gene distributions across the bacteriophage isolates. The *Escherichia* phages exhibited notable similarity, each containing 289 coding sequences (CDS) with a coding density of 95.83% and genome lengths ranging from 169,756 to 171,449 base pairs. These isolates consistently encoded four tRNA genes, three DNA connector genes, 52 to 54 metabolism-related genes, 18 head genes, 29 tail genes, 10 auxiliary metabolic genes, seven to eight lysis genes, and 10 transcription regulation genes (Figure 4). In contrast, the *S. boydii* phages displayed 280 to 286 CDS with coding densities between 95.86% and 96.07%, featuring variable tRNA content (three to 11 genes) and similar structural gene distributions (Figure 4). The *R. ornithinolytica* phages demonstrated a consistent architecture with 295 CDS each, elevated tRNA content (17 genes), and characteristic structural gene patterns (Figure 4). Notably, Phage_Ob_P presented a compact genome architecture with 177 CDS, reduced structural gene content (six head genes, 13 tail genes), and the highest. Importantly, none of the isolates contained CRISPRs, integration/excision genes, tmRNAs, VFDB virulence factors, or CARD antimicrobial resistance genes, thereby supporting their potential safety profile for therapeutic applications, though experimental confirmation is warranted, as represented in Figure 4. The genomic characterisation of the eight bacteriophage isolates collectively revealed a diverse yet functionally coherent set of phages with strong potential for therapeutic and biocontrol applications.

### 3.7. Gene Prediction Tool Performance Across Bacteriophage Genomes

The detailed per-phage evaluation of seven gene prediction algorithm performance and consensus patterns across all eight bacteriophage genomes is provided in Supplementary Materials (Table S3, Figures S11–S18). Briefly, consensus rates varied from 9.2% to 18.3%, with Decouphage consistently contributing the highest unique predictions.

### 3.8. Bacteriophages Comparative Genomic Analysis Using CompareM

**Figure 5:**
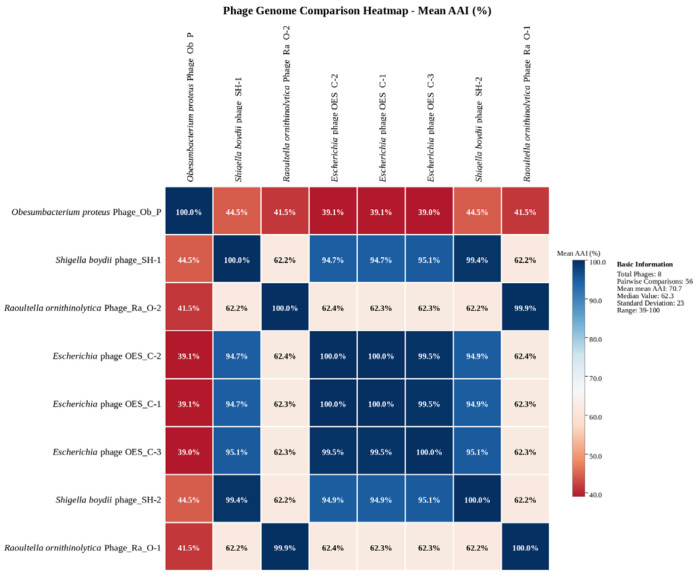
This heatmap illustrates the pairwise mean amino acid identity (AAI) percentages among eight bacteriophage isolates, offering insights into their evolutionary relationships and genomic conservation. The analysis identified three distinct clusters of highly related phages with exceptional genomic similarity: (1) Phage OES_C-1, Phage OES_C-2, and Phage OES_C-3 form a closely related clade with 99.5–100% AAI, suggesting they represent nearly identical or recently diverged strains; (2) Phage_SH-1 and Phage_SH-2 exhibit 99.4% AAI, indicating they are quasi-identical isolates; and (3) Phage_Ra_O-1 and Phage_Ra_O-2 share 99.9% AAI, representing another pair of highly conserved phages. Cross-genus comparisons reveal that *Escherichia* and *Shigella* phages share substantially high AAI values (94.7–95.1%), reflecting the close phylogenetic relationship between these bacterial genera and suggesting these phages likely share recent common ancestry and conserved structural proteins. The *Raoultella* phages display moderate AAI (62.2–62.4%) with both *Escherichia* and *Shigella* phages, indicating more distant yet appreciable evolutionary relationships. In stark contrast, Phage_Ob_P exhibited markedly low AAI values (39.0–44.5%) with all other isolates, positioning it as a phylogenetically distinct lineage with minimal amino acid sequence conservation, likely representing a different viral family or subfamily.

Comparative genomic analysis conducted using CompareM elucidated distinct evolutionary relationships among the eight bacteriophage isolates. The mean amino acid identity (AAI) heatmap revealed clear clustering patterns: phages OES_C-1, OES_C-2, and OES_C-3 form a highly cohesive group with AAI values exceeding 90%, indicating a recent common ancestry and suggesting they may represent variants of the same species. Phage_SH-1 and Phage_SH-2, along with Phage_Ra_O-1 and Phage_Ra_O-2, exhibit moderate similarity (60–80% AAI), positioning them as intermediate groups with a shared evolutionary history but sufficient divergence to constitute distinct lineages. Phage_Ob_P is a notable outlier, displaying consistently low AAI values (below 50%) and minimal orthologous gene overlap, suggesting it represents a highly divergent evolutionary branch (Figure 5). The orthologous gene count matrix indicated the highest shared gene numbers (275) between closely related *Escherichia* and *Raoultella* phages, while Phage_Ob_P consistently exhibits the lowest counts (137–149) across all comparisons (Figure S19). Despite infecting related Enterobacteriaceae hosts, these phages have followed distinct evolutionary trajectories shaped by host-specific pressures, ecological niches, and varying horizontal gene transfer rates, resulting in mosaic genomic architectures.

### 3.9. Terminase Large Subunit-Based Phylogenetic Analysis of Bacteriophages

**Figure 6:**
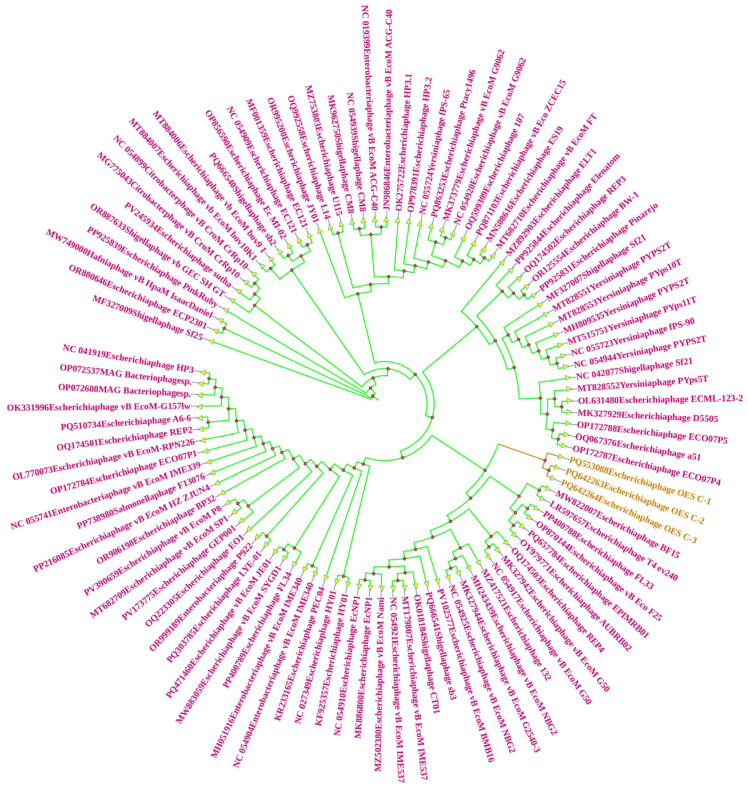
The circular phylogenetic tree depicts the evolutionary relationships among Phages OES_C-1, OES_C-2, and OES_C-3, alongside related bacteriophages, based on the amino acid sequences of the terminase large subunit (TerL). The tree delineates distinct phylogenetic groupings through color-coded branches, each representing different evolutionary lineages. The three OES phages constitute a monophyletic clade, indicated by orange and yellow branches, with Phage OES_C-1 occupying a basal position and Phage OES_C-2 and Phage OES_C-3 forming a derived sister pair. This OES clade predominantly clusters with phages infecting *Escherichia* and *Shigella* species, forming a compact monophyletic group characterized by short branch lengths, which suggest recent divergence from a common ancestor and significant sequence similarity.

Through a comprehensive phylogenetic analysis of terminase large subunit sequences among enterobacterial phages, this study elucidated complex evolutionary relationships that transcend traditional host–pathogen boundaries within the Enterobacteriaceae family. Phylogenetic analysis of the terminase large subunit reveals that Phages OES_C-1, OES_C-2, and OES_C-3 form a distinct monophyletic clade within the Myoviridae family. Within this clade, OES_C-1 occupies a basal position, representing the ancestral form, while OES_C-2 and OES_C-3 constitute a recently diverged sister pair within a derived subclade. This evolutionary pattern, characterized by the initial divergence of OES_C-1 followed by the speciation of OES_C-2 and OES_C-3, suggests adaptive responses to specific selective pressures or host-associated factors. The OES clade emerges from a complex polytomy that encompasses a variety of T4-like phages, including *Escherichia* phage T4_ev240 (LR597657), vB_EcoM morphotypes (G50, NBG2, IME537), *Shigella* phages, and extends to *Yersinia* phages (PYPS2T, PYps5T) and *Salmonella* phage F13076. This situates the OES series within the broader enterobacterial phage radiation while maintaining distinct terminase characteristics that affirm their monophyletic status (Figure 6). The *Shigella* phages SH-1 and SH-2 demonstrate notable evolutionary plasticity, clustering within *Escherichia*-dominated clades rather than exclusively with *Shigella* phages. Phage_SH-1 exhibits the closest affinity to *Escherichia* phages, including REP4, vB_EcoM_G50, and vB_EcoM_NBG2, while Phage_SH-2 forms a sister relationship with *Escherichia* phage PinkRuby (Figures S20 and S21). This cross-genus clustering suggests that host range plasticity and horizontal gene transfer have played a more significant role than strict host phylogeny in shaping terminase evolution. The *Raoultella* phages occupy distinct positions, indicating independent evolutionary trajectories. Phage_Ra_O-1 clusters with *Klebsiella* phage KP01, whereas Phage_Ra_O-2 resides in a different subclade with *Klebsiella* Phages vB_KpnM_GF and vB_Kpn_AM_K3 (Figures S22 and S23). Both demonstrate closer relationships with *Klebsiella* phages than with each other, implying that functional constraints have superseded host phylogeny. Phage_Ob_P occupies a unique niche, clustering with *Salmonella* phage vB_SenS_UTK0010 and *Escherichia* phage Spoonbill Gilly (Figure S24). This positioning suggests recent diversification, potentially reflecting adaptation to the specific ecological niche of *O. proteus* in brewery environments, with conserved DNA packaging mechanisms but divergent host recognition systems. Collectively, the phylogenetic results indicated that the evolution of terminase among the studied phages has been primarily driven by horizontal gene transfer, adaptability in host range, and ecological specialization, rather than being confined by strict host phylogenetic limits. The observed cross-genus clustering patterns across all isolates align with their experimentally confirmed broad-spectrum infectivity, further supporting their potential as adaptable biological control agents against a variety of Enterobacteriaceae hosts.

### 3.10. Bacteriophage DeePVP Protein Classification Analysis

**Figure 7:**
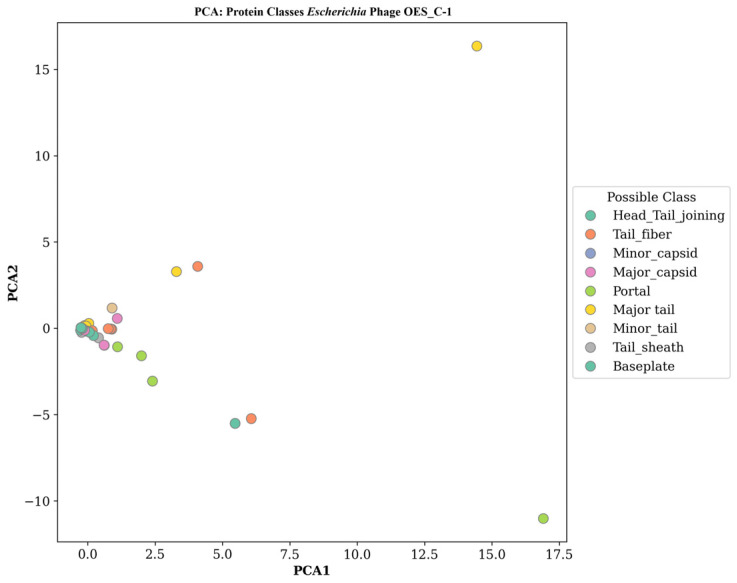
Principal Component Analysis (PCA) of Phage OES_C-1 structural proteins reveals distinct functional clustering patterns. The analysis identified distinct clustering patterns that reflect functional and structural diversity. Tail fiber proteins exhibit notable dispersion, with one positioned distantly from others, indicating highly distinct characteristics likely related to specialized host recognition domains or unique amino acid composition. Head-tail joining proteins and minor capsid proteins cluster together, suggesting shared biochemical properties such as hydrophobicity, charge distribution, or secondary structure. The major capsid protein groups tightly near the origin, reflecting conserved characteristics typical of structural scaffolding proteins. Portal, major tail, and tail sheath proteins display moderate dispersion, reflecting unique structural requirements for tail assembly components that form extended multimeric structures. The baseplate protein clusters near the origin, sharing biochemical characteristics with core structural components.

DeePVP-based protein classification was performed for all eight bacteriophage isolates. Principal component analysis of structural proteins across phage isolates revealed distinct functional groupings that reflect the modular architecture of virions, characterized by both conserved and specialized features. Initially, Phage OES_C-1 demonstrated a tight clustering of head and capsid-associated proteins (major capsid, minor capsid, head-tail joining) near the origin, indicating a high degree of structural conservation for capsid stability and genomic protection. In contrast, tail-associated proteins (tail fiber, minor tail, major tail, tail sheath) exhibit greater heterogeneity, reflecting functional complexity for host recognition and DNA injection. Portal and baseplate proteins occupy intermediate positions as structural interfaces, with one major tail protein variant appearing as a significant outlier, suggesting specialized host cell penetration (Figure 7). Similarly, Phage OES_C-2 displayed functional groupings with notable structural diversification. Head and capsid proteins cluster near the origin with moderate dispersion, indicating evolutionary adaptation for capsid assembly, while tail-associated proteins exhibit remarkable heterogeneity. Major tail proteins are located at extreme outlier coordinates (approximately 3,17 and 17, −11), indicating significant functional specialization, and portal proteins demonstrate structural divergence, suggesting diversified DNA packaging mechanisms (Figure S25). Likewise, Phage OES_C-3 showed tight clustering of head and capsid proteins, indicating high conservation, while tail-associated proteins display characteristic diversification. Major tail proteins are found at distant coordinates (approximately 5,1.5 and 17,15.5), suggesting evolutionary adaptation for mechanical functions, and portal proteins display extreme outliers (approximately 17, −12.5), indicating unique DNA packaging specialization (Figure S26).

The *Shigella* phages revealed host-specific adaptations: Phage_SH-1 displayed major capsid outliers (approximately 17, −10.5), indicating adaptation to the *Shigella* cellular environment, with major tail protein heterogeneity (coordinates 3, 0.5 and 17, 15), suggesting evolutionary adaptation for host penetration (Figure S27). Phage_SH-2 exhibited comparable patterns, with major capsid and major tail protein outliers (17, −10.5 and 17, 15) and tail fiber proteins showing moderate heterogeneity reflecting complex *Shigella* surface antigen diversity (Figure S28). The *Raoultella* phages demonstrated additional architectural complexity through collar protein inclusion: both Phage_Ra_O-1 and Phage_Ra_O-2 exhibited tight head, capsid, and collar protein clustering, indicating structural conservation, while tail-associated proteins showed diversification with major tail proteins at dispersed coordinates and minor tail proteins exhibiting remarkable heterogeneity (Figures S29 and S30). Phage_Ob_P revealed distinct organizational patterns, with tight head and capsid protein clustering contrasting with remarkable tail fiber diversification (approximately 0.5, 1.5; 0.5, 3; and 8, 17) and an extreme portal protein outlier (approximately 18, −7), indicating unique DNA packaging mechanisms adapted to *Obesumbacterium* hosts (Figure S31).

The principal component analysis (PCA) of structural proteins from all eight bacteriophage isolates collectively demonstrated a consistent architectural pattern: conserved head and capsid proteins are contrasted by highly varied tail-associated proteins. This pattern reflects an evolutionary balance between maintaining structural stability and achieving functional adaptability. The frequent occurrence of major tail, minor tail, and portal protein anomalies across different isolates highlights the influence of positive selection in diversifying mechanisms for host recognition and DNA packaging. Additionally, the presence of collar proteins in *Raoultella* phages points to lineage-specific architectural innovations. These patterns of structural diversification align with the broad host range and cross-species infectivity that have been experimentally observed in these phages, further supporting their potential as adaptable biocontrol agents.

**Figure 8:**
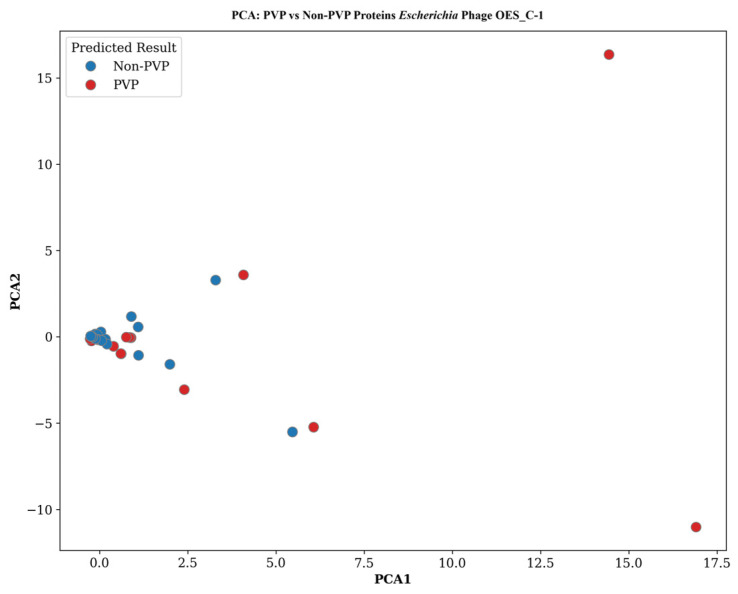
illustrates the results of a principal component analysis, which reveals distinct clustering patterns between putative virion proteins (PVP) and non-PVP proteins in Phage OES C-1. The majority of proteins from both categories cluster near the origin, suggesting shared physicochemical properties. However, three PVP outliers located at extreme coordinates likely represent specialized structural components, such as tail fibers or baseplate proteins, indicative of the functional diversification characteristic of myoviridae architecture. Additionally, a single non-PVP outlier suggests a protein with specialized regulatory or lytic functions. This distribution demonstrates that while most phage proteins exhibit common features, certain PVP proteins possess distinctive characteristics that correlate with their specialized structural roles in virion assembly.

The principal component analysis of predicted virion proteins (PVP) versus non-PVP proteins reveals distinct clustering patterns that reflect conserved molecular distinctions and host-specific adaptations. The OES phage series demonstrated a tight clustering of non-PVP proteins in the lower-left quadrant, suggesting shared physicochemical properties consistent with cytoplasmic or enzymatic functions. In contrast, PVP proteins exhibit a dispersed distribution with distinct outliers at coordinates (15,17) and (17, −11) in Phage OES_C-1, indicating substantial heterogeneity in structural components (Figure 8). Phage OES_C-2 showed remarkably consistent patterns, with non-PVP clustering between (0–3, −2 to +2) and PVP outliers at similar coordinates (15,17 and 17, −11), suggesting evolutionarily conserved molecular determinants (Figure S32). Phage OES_C-3 displayed a distinctive deviation, with more dispersed non-PVP distribution and reduced PVP outliers, with only two proteins at distant positions (approximately 17,16 and 17, −12), potentially indicating evolutionary divergence in the structural protein repertoire (Figure S33). The *Shigella* phages exhibit intermediate clustering patterns that bridge classical *Escherichia* distributions and unconventional profiles. Phage_SH-1 showed tight non-PVP clustering with moderate dispersion to coordinates (3,2) and (5, −5) and reduced PVP outliers at distant coordinates (17,15) and (17, −10), with several clustering proximally, including one at (5, −8) (Figure S34). Phage_SH-2 demonstrated remarkable consistency, with identical non-PVP clustering to coordinates (4,1) and (5, −5) and precisely mirroring PVP distribution with outliers at (17,15) and (17, −11), alongside intermediate proteins at (5, −8), suggesting an evolutionarily optimized *Shigella boydii* infection architecture (Figure S35). In stark contrast, the *Raoultella* phages presented striking departures characterized by a complete absence of predicted PVP proteins and unusually broad non-PVP distribution. Phage_Ra_O-1 displayed extensive dispersion from tightly clustered populations to extreme outliers (12,16; 18, −3; and 10, −7), suggesting extraordinary functional diversity indicative of highly specialized or evolutionarily divergent lineages employing novel virion assembly mechanisms (Figure S36). Phage_Ra_O-2 exhibited a remarkably similar distribution, with complete PVP absence and broad non-PVP dispersion to coordinates (10,15.5; 19, −2; and 8.5, −6.5), reinforcing this unconventional architecture as a conserved feature of *Raoultella*-infecting phages (Figure S37). Finally, Phage_Ob_P revealed a fundamentally different protein clustering architecture, with non-PVP proteins maintaining characteristic tight clustering but exhibiting greater dispersion with outliers extending to coordinates (7.5, −4) and (18, −7), suggesting more diverse physicochemical properties. Meanwhile, PVP proteins display a markedly altered distribution, with the majority clustering closely with non-PVP proteins and only a single extreme outlier at approximately (8, 17) (Figure S38). The analysis of clustering between PVP and non-PVP proteins collectively indicated that, although most isolates retain conserved physicochemical differences between structural and non-structural proteins, there are notable deviations specific to hosts and lineages. Non-PVP proteins consistently form tight clusters across the OES and *Shigella* phage series, which starkly contrasts with the complete lack of predicted PVP proteins in *Raoultella* phages. This suggests fundamentally different virion assembly strategies that may represent new evolutionary lineages. The varied distribution of PVPs observed in Phage_Ob_P further emphasizes the architectural diversity among these isolates. These results collectively underscore the molecular heterogeneity that supports the wide host range of these phages and affirm the value of PVP-based proteomic profiling as a tool for distinguishing and characterizing phages functionally.

### 3.11. SDS-PAGE Analysis of Bacteriophage Protein Profiles

**Figure 9:**
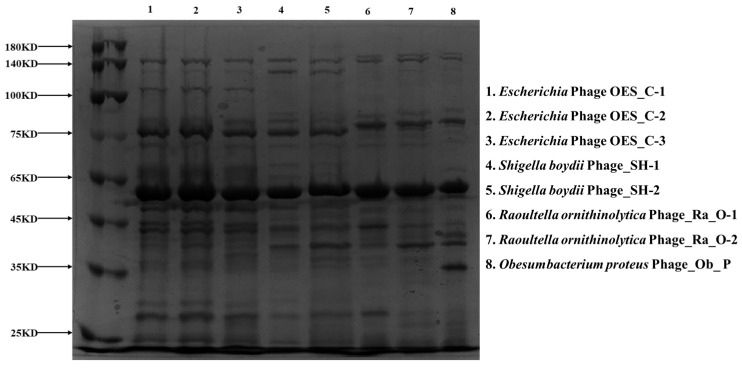
The SDS-PAGE gel electrophoresis illustrates the protein profiles of purified bacteriophages, separated according to molecular weight. The first lane contains the protein molecular weight marker, with sizes indicated from 180 to 25 kDa. The gel reveals distinct protein banding patterns for each phage, with prominent bands corresponding to major structural proteins, including major capsid proteins in the approximately 55–65 kDa region, and various tail-associated proteins distributed across the molecular weight range.

SDS-PAGE analysis of eight bacteriophage proteomes revealed distinct protein banding patterns that strongly correlated with Pharokka genome annotations, facilitating precise protein identification based on molecular weight. Phages OES_C-1, OES_C-2, OES_C-3, SH-1, SH-2, Ra_O-1, and Ra_O-2 exhibited highly similar T4-like profiles, with prominent bands corresponding to major structural proteins: tail sheath (71.38–71.76 kDa), major head (55.93–56.26 kDa), portal (60.75–61.08 kDa), and DNA polymerase (103.58–104.69 kDa). Variations in molecular weight were observed in host-specificity determinants, with *Raoultella* phages displaying larger tail fiber proteins (160.17–160.85 kDa) compared to *Escherichia* and *Shigella* phages (139.95–140.55 kDa), reflecting host adaptation. In contrast, Phage_Ob_P exhibited a markedly different profile with a smaller major head protein (50.63 kDa), and a characteristic base plate tail tube protein (34.51 kDa) absent in T4-like phages, indicating membership in a different phage family. Band intensities correlated with predicted gene expression levels, with DNA replication machinery (terminase large subunit 69.72–69.84 kDa, DNA topoisomerase II 68.31–68.81 kDa) and metabolic enzymes (ribonucleotide reductase 84.80–91.97 kDa) appearing as prominent bands (Figure 9). These findings validated computational predictions through experimental proteomics and demonstrated the utility of SDS-PAGE for distinguishing bacteriophage families based on characteristic structural protein profiles.

### 3.12. KEGG Virion Assembly Pathway Analysis

**Figure 10:**
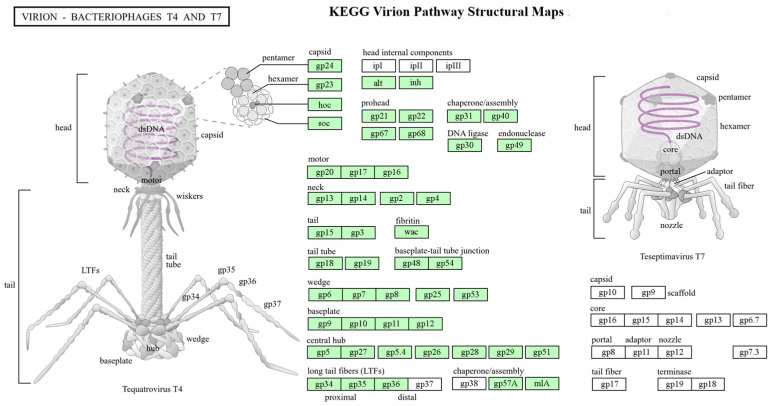
presents a comparative diagram delineating the protein structural pathway of Phages OES_C-1 and OES_C-2, with bacteriophage T4 serving as a reference model. Both phages exhibit an identical structural organization, with genes hierarchically arranged from head to tail as follows: capsid assembly (gp24 pentamer, gp23 hexamer, hoc, soc), head internal components (alt, inh), prohead formation (gp21, gp22, gp67, gp68), chaperone and assembly factors (gp31, gp40), enzymatic components (gp30 DNA ligase, gp49 endonuclease), DNA packaging motor (gp20, gp17, gp16), neck structure (gp13, gp14, gp2, gp4), tail sheath (gp15, gp3), fibritin (wac), tail tube (gp18, gp19), baseplate-tail tube junction (gp48, gp54), wedge proteins (gp6, gp7, gp8, gp25, gp53), baseplate assembly (gp9, gp10, gp11, gp12), central hub (gp5, gp27, gp5.4, gp26, gp28, gp29, gp51), and long tail fiber assembly with proximal and distal fibers (gp34, gp35, gp36), facilitated by chaperone factors (gp57A, mlA).

A comparative analysis of virion assembly pathways was performed across seven T4-like bacteriophages infecting Enterobacteriaceae, using bacteriophage T4 as a reference model, revealing both conserved structural modules and notable host-specific variations in protein composition. All isolates shared a common structural framework encompassing capsid assembly (gp24 pentamer, gp23 hexamer, hoc, soc), prohead formation machinery (gp21, gp22, gp67, gp68), DNA packaging motor (gp20, gp17, gp16), neck structure (gp13, gp14, gp2, gp4), and baseplate complex components. However, significant differences were identified across isolates. Phages OES_C-1 and OES_C-2 displayed a core T4-like architecture with standard head internal proteins (alt, inh) and a three-component long tail fiber assembly (gp34, gp35, gp36) (Figure 10). Phages OES_C-3 and SH-1 presented an expanded head internal protein complement including ipI, while incorporating an additional gp37 tail fiber component, suggesting enhanced host recognition capability (Figure S39). Phage_SH-2 exhibited the most comprehensive structural organisation, possessing the complete head internal protein repertoire (ipI, ipII, alt, inh) and full four-component tail fiber assembly (gp34-gp37), reflecting its superior infection architecture adapted to *S. boydii* (Figure S40). In contrast, *Raoultella* phages Ra_O-1 and Ra_O-2 demonstrated a streamlined architecture characterised by the absence of hoc, a simplified tail sheath lacking gp3 and fibritin protein wac, a reduced baseplate assembly missing gp12 and gp28, and a simplified two-component tail fiber system (gp34, gp36), representing the most structurally economical configuration among all isolates (Figure S41). Notably, Phage_Ob_P yielded no KEGG pathway annotation, potentially reflecting genuine genomic divergence or current limitations in annotation databases for this phage-host system, warranting further investigation. The comparative analysis of virion assembly revealed that although all characterized phages possess a conserved T4-like structural framework, there is notable modularity in the composition of head internal proteins, the complexity of tail fibers, and the architecture of the baseplate, which reflects evolutionary adaptations specific to their hosts. The increasing complexity observed from the simplified *Raoultella* phages to the more intricate Phage_SH-2 highlights the influence of host-driven selection on virion assembly strategies, especially in tail fiber systems that facilitate host recognition. These structural differences align with the varied adsorption efficiencies and host range profiles demonstrated experimentally across isolates, emphasizing the connection between virion architecture and the infection versatility of these bacteriophages.

## 4. Discussion

The eight bacteriophage isolates examined in this research exhibited strictly lytic lifestyles, a wide host range infectivity among phylogenetically diverse Enterobacteriaceae, high adsorption rates, favorable plating efficiency values, and genomically verified safety profiles, characterized by the absence of virulence, antimicrobial resistance, and lysogeny-related genes, along with conserved T4-like virion structures with host-specific structural modifications. These features collectively meet the essential criteria set for phage candidates intended for therapeutic and biocontrol purposes [56]. Such traits align with previously established standards for phage suitability in food safety and clinical settings, where obligate lytic activity, broad-spectrum infectivity, and genomic biosafety are considered essential qualities for effective biological control agents [9,57].

In the current study, the isolation of Enterobacteriaceae from raw milk samples, predominantly comprising *E. coli* (40%) and *Shigella boydii* (33%), highlights ongoing food safety challenges that align with global contamination rates of 15–60% [4,58]. The low infectious dose of *S. boydii* (10–100 cells) and its increasing antimicrobial resistance are particularly concerning [59,60]. The detection of *R. ornithinolytica* (13.3%), *O. proteus*, and S. sonnei raises emerging concerns due to their clinical significance, environmental persistence, and potential for nosocomial infections [61,62,63,64]. Prophage induction experiments utilizing Mitomycin C (0.5 and 10 μg/mL) yielded negative results, despite its known activation of the SOS response pathway, suggesting either the absence of integrated prophages or resistance to induction [65,66].

Eight bacteriophage isolates were successfully recovered from environmental wastewater sources, displaying large, clear lytic zones with pinpoint plaque morphology, indicative of efficient lysis and potential depolymerase activity [9,67,68,69,70].

Transmission electron microscopy confirmed these isolates as members of the Myoviridae family, characterized by icosahedral heads (99–118 nm in diameter) and contractile tails (95–125 nm in length), aligning with established therapeutic phage dimensions and safety profiles [11,13,71,72].

The growth dynamics of eight bacteriophage isolates revealed that phylogenetic relatedness does not predict infection efficiency. *E. coli* served as a highly permissive host for both homologous and heterologous phages, with *Shigella* phages achieving titers of 1.25 × 10^11^ to 1.65 × 10^11^ PFU despite cross-genus infection [73,74]. Paradoxically, native *Raoultella* phages exhibited attenuated replication (2 × 10^9^ to 2.50 × 10^9^ PFU) compared to heterologous phages (up to 7 × 10^10^ PFU), potentially indicating strain-specific resistance mechanisms [75,76]. Burst sizes varied considerably (11–166 particles/cell), reflecting differences in host metabolic capacity and virion assembly efficiency [77,78]. Extended latent periods for heterologous infections (60–100 min versus 30–40 min for homologous infections) likely reflect delays in receptor binding kinetics [79]. Notably, heterologous phages performed exceptionally on *O. proteus*, where *Raoultella* phage achieved 2.5-fold higher titers than native phage with burst sizes exceeding 130 particles/cell, challenging assumptions about co-evolved phage-host pairs [80,81]. Biphasic growth kinetics observed with certain phage-host combinations may indicate sequential infection rounds or phage-induced metabolic reprogramming [82,83].

Adsorption kinetics across four Enterobacteriaceae hosts revealed efficient receptor-binding, with efficiencies ranging from 84.75% to 99.98% and maximum rates reaching 1.88 × 10^−12^ within 10 min, demonstrating the classical biphasic pattern of tailed bacteriophages [79,84]. Notably, heterologous phages achieved adsorption efficiencies comparable to or exceeding homologous phages, with Phage_Ob_P on *E. coli* (99.60%) and *Raoultella* phages on *S. boydii* (99.67%) suggesting tail fiber architectures that recognize conserved receptor motifs across related genera [12,85]. *Shigella* phages exhibited exceptionally high adsorption on their native host (99.93–99.98%), reflecting extensive surface antigen conservation between *Escherichia* and *Shigella*, which share lipopolysaccharide structures and outer membrane proteins [86,87]. Strong correlations between adsorption rate and velocity parameters (R^2^ > 0.92–0.95) confirmed first-order kinetics, while temporal deceleration beyond 10 min indicated receptor saturation [78]. The rapid initial phase accounted for 70–87.5% of total binding within 10 min, consistent with diffusion-limited collision models [88,89]. Variable adsorption efficiencies among *Escherichia* phages on *S. boydii* (90–98%) suggest that subtle variations in lipopolysaccharide structures or outer membrane proteins significantly influence receptor accessibility, emphasizing that host range determination extends beyond receptor presence to include density and spatial distribution [90,91].

Host range analysis across 21 bacterial strains revealed significant heterogeneity, with Phage_SH-2 exhibiting the broadest infectivity (95.2%) and Phage_Ob_P the most restricted (47.6%), suggesting that host range depends on tail fiber architecture, receptor diversity, and defense mechanisms rather than taxonomic relatedness [12,91]. *E. coli* served as a universal permissive host, supporting all eight phages with efficiency of plating (EOP) values of 1.0–25.0, reflecting absent restriction-modification systems in this laboratory strain [92,93]. Paradoxically, *S. flexneri* KS7 showed higher susceptibility to heterologous *Escherichia* phages (EOP 1.2–3.16) than homologous *Shigella* phages (EOP 0.08–0.2), suggesting receptor polymorphisms from horizontal gene transfer altered surface architectures [76,94]. Dramatic strain-level variation, exemplified by *E. coli* Z22–7’s susceptibility to Phage_Ob_P (EOP 4.17) while other *E. coli* strains remained resistant (EOP 0.0–0.2), demonstrates that species-level taxonomy insufficiently predicts infectivity [73,95]. Native *Raoultella* phages showed restricted host range, with Phage_Ra_O-1 achieving high-efficiency infection only on its homologous host (EOP 4.12), suggesting evolutionary trade-offs between range breadth and infection efficiency [96,97]. High susceptibility of phylogenetically distant *K. variicola* to Phage_Ra_O-2 (EOP 4.0) indicates conserved receptor structures enabling cross-genus infection [98,99]. General resistance of *S. sonnei* and most *S. flexneri* strains, with notable exceptions such as MCURPq_BL04’s susceptibility to Phage_Ra_O-2 (EOP 2.4), underscores the unpredictable nature of phage-host interactions [100,101].

Genomic analysis has identified significant architectural diversity (103–170 kb) characteristic of Myoviridae genomes (100–200 kb), which encode 150–300 proteins [71,102,103]. Notably, there is a complete absence of antimicrobial resistance genes, virulence factors, and CRISPR elements, thereby providing exceptional safety assurance for therapeutic applications [104,105]. The BACPHLIP lifestyle prediction confirmed strictly lytic characteristics, with virulent scores ranging from 0.9801 to 1.0, thus eliminating concerns regarding lysogenic integration, which is critical for therapeutic phage applications. This suggests an environmental selection favoring lytic populations [106,107].

Comprehensive annotation revealed coding densities of 92.27–96.07%, with a substantial number of DNA/RNA/nucleotide metabolism genes (47–55 per genome), underscoring metabolic independence [11,108]. Consistent structural components were identified, including 6–18 head/packaging genes and 13–29 tail genes, alongside a high proportion of genes of unknown function (104–164, approximately 50–55%), representing viral genomic “dark matter” [109]. The absence of CRISPRs, integration/excision genes, VFDB virulence factors, and CARD antimicrobial resistance genes further confirmed the therapeutic safety profiles [100,110].

Comparative analysis of seven gene prediction algorithms revealed substantial inter-tool variability, with consensus rates of 9.2–18.3%, reflecting atypical phage genomic characteristics such as unusual codon usage, overlapping genes, and alternative genetic codes [39,40,41,42,43,111]. Individual tools contributed 28–293 unique predictions beyond the core consensus [42], with Glimmer showing pronounced degradation [40,112]. Host-dependent patterns exhibited lower consensus in *Shigella* and *Raoultella* phages, suggesting effects of phylogenetic distance [113,114]. The compact *Obesumbacterium* phage (230 genes) highlighted challenges in annotating small genomes [14,115].

CompareM analysis elucidated intricate evolutionary relationships, with *Escherichia* phages forming cohesive groups characterized by greater than 90% average amino acid identity (AAI). In contrast, the *Obesumbacterium* phage emerged as an evolutionary outlier with less than 50% AAI. Intermediate similarity was observed in *Shigella* and *Raoultella* phages, with AAI ranging from 60% to 80%, reflecting taxonomic ambiguities and frameworks for viral species delineation, where an AAI exceeding 95% indicates conspecific relationships, and an AAI below 60% suggests distinct species [72,116]. The pronounced divergence of Phage_Ob_P, characterized by 137–149 orthologous genes and an orthologous fraction of less than 30%, suggests ancient divergence or adaptation to ecological niches, consistent with host-specific selective pressures [117,118]. Terminase-based phylogenetic reconstruction demonstrated that functional constraints and horizontal gene transfer supersede strict bacterial phylogeny in shaping phage evolution. This is evidenced by the cross-genus clustering of *S. boydii* phages within *Escherichia*-dominated clades, independent trajectories of *R. ornithinolytica* phages clustering with *Klebsiella* lineages, and the positioning of *O. proteus* phages within Salmonella and *Escherichia* clades, exemplifying evolutionary flexibility and convergent evolution of DNA packaging mechanisms [102,119,120]. The conclusion that HGT has driven terminase evolution is supported by three independent lines of evidence: (i) the incongruence between terminase-based phylogeny and host taxonomy (e.g., *Shigella* phages clustering within *Escherichia* clades despite their distinct bacterial hosts); (ii) the mosaic genomic structures identified by comparative genomics, in which conserved structural modules are flanked by genome-specific sequence blocks of divergent origin; and (iii) the experimentally confirmed broad host range, mechanistically consistent with gene-level acquisition of novel tail fiber or receptor-binding domain variants via HGT. While direct demonstration of individual HGT events would require longitudinal sampling and functional validation of acquired genes, the convergent topological patterns across multiple independent phylogenetic clusters constitute robust circumstantial evidence for HGT as a primary evolutionary driver [117,118,119].

DeePVP protein classification revealed distinct molecular signatures between phage virion proteins (PVPs) and non-PVPs, supporting advances in machine learning [51,121]. Non-PVP clustering was attributed to cytoplasmic functions, while PVPs exhibited heterogeneity reflecting diverse roles in virion assembly and host recognition [122,123]. Conserved head and capsid proteins contrasted with diverse tail proteins due to host-specific adaptation [124,125]. Notably, the absence of predicted PVPs in *R. ornithinolytica* phages suggests novel assembly mechanisms or reductive evolution, challenging traditional schemes [126,127,128,129].

SDS-PAGE analysis corroborated computational annotations, with experimental molecular weights aligning with Pharokka predictions [130]. This analysis revealed highly similar profiles among phages (OES_C-1, OES_C-2, OES_C-3), phages (SH-1, SH-2), and phages (Ra_O-1, Ra_O-2), supporting their classification as T4-like through conserved structural proteins, including the tail sheath (71.38–71.76 kDa), major head protein (55.93–56.26 kDa), portal protein (60.75–61.08 kDa), and DNA polymerase (103.58–104.69 kDa) [100,130,131]. Variations in tail fiber molecular weights between *Raoultella* phages (160.17–160.85 kDa) and *Escherichia* and *Shigella* phages (139.95–140.55 kDa) reflect host-range determinant evolution and modular phage evolution [132,133,134]. In contrast, Phage_Ob_P’s distinct profile, with a smaller major head protein (50.63 kDa) and a characteristic base plate tail tube protein (34.51 kDa), confirmed its taxonomic distinction and alternative family membership [13,71].

The remarkable conservation of T4 virion assembly pathways across seven isolates from phylogenetically diverse Enterobacteriaceae hosts demonstrates the evolutionary optimization of this structural framework’s efficiency in particle formation and host exploitation [11,134]. Identical gene repertoires for structural and enzymatic components, including three independent assembly pathways (head, tail, long tail fibers), reflect a sophisticated modular architecture [135,136]. Preserved major structural proteins (gp23, gp24), accessory proteins (hoc, soc), head internal components (ipI, ipII, ipIII), prohead chaperones (gp21, gp22), neck/motor assembly components (gp20, gp17, gp16), baseplate complex (15 gene products, 13 assembly steps), and enzymatic components (DNA ligase gp30, endonuclease gp49) indicate fundamental mechanisms under strong selective pressure for structural integrity, proper DNA packaging, ATP-dependent translocation, host recognition, and genome processing maintained across different enterobacterial cellular environments [89,108,137,138,139]. This demonstrates that convergent evolution or retention of T4/T7-type strategies represents optimal performance characteristics for a lytic lifestyle, despite rapid host adaptation capabilities [11,134].

Remarkably, Phage_Ob_P consistently stood out as an unusual isolate across various independent analytical methods used in this research. Although it shares similar morphological traits, such as an icosahedral head and a contractile tail, and can infect hosts within the Enterobacteriaceae family, Phage_Ob_P exhibited significantly different genomic and proteomic profiles compared to the other seven isolates. Genomic analysis using CompareM showed amino acid identity values below 50% and the least overlap in orthologous genes (137–149 shared genes; orthologous fraction < 30%) in all pairwise comparisons, marking this phage as a distinct evolutionary anomaly. This divergence was further confirmed by terminase-based phylogenetic analysis, SDS-PAGE proteomic profiling which identified a unique structural protein composition, including a smaller major head protein (50.63 kDa) and a distinct baseplate tail tube protein (34.51 kDa) and DeePVP analysis, which indicated an unusual portal protein distribution suggesting different DNA packaging mechanisms. Additionally, Phage_Ob_P was the only isolate for which KEGG pathway annotation did not identify any virion assembly pathway, possibly due to genuine genomic novelty or current gaps in reference databases for phages infecting *Obesumbacterium*. The collective evidence from genomic, phylogenetic, proteomic, and functional analyses strongly indicates that Phage_Ob_P represents a unique evolutionary branch that deserves further study beyond this research. Importantly, to the best of our knowledge, Phage_Ob_P constitutes the first bacteriophage ever isolated and characterized against *O. proteus*, as no prior report of a phage infecting this host has been identified in the published literature or in publicly available genomic databases including NCBI GenBank, underscoring the novelty of this isolate and the significance of its further characterization. Future investigations using cryo-electron microscopy, broader metagenomic sampling, and host-range evolution experiments are recommended to understand the structural and functional reasons for its divergence and to determine its taxonomic position within the broader group of Enterobacteriaceae-infecting phages.

## 5. Conclusions

This study provided a comprehensive biological, genomic, and proteomic characterization of eight novel bacteriophages isolated from wastewater, targeting foodborne Enterobacteriaceae of both clinical and industrial importance. Each of the eight isolates exhibited a strictly lytic lifestyle, as confirmed by BACPHLIP predictions with virulent scores ranging from 0.975 to 1.0. They displayed Myoviridae morphology, characterized by icosahedral heads measuring 99–118 nm and contractile tails of 95–125 nm, along with a broad-spectrum infectivity across 21 bacterial strains, ranging from 47.6% to 95.2%. The phages also showed rapid adsorption efficiencies, reaching up to 99.98%. Whole-genome sequencing indicated genome sizes between 103 and 170 kb with high coding densities of 92% to 96%. Importantly, none of the isolates contained antimicrobial resistance genes, virulence factors, CRISPR elements, or lysogeny-associated genes, thereby meeting the essential biosafety standards for therapeutic and biocontrol uses. Comparative genomic and phylogenetic studies revealed that phage evolution within Enterobacteriaceae is largely influenced by horizontal gene transfer and host-range adaptability, rather than strict bacterial phylogeny, as demonstrated by cross-genus clustering patterns and mosaic genomic structures.

The results collectively indicate that the identified phages are potential candidates for developing phage-based biocontrol strategies aimed at combating foodborne Enterobacteriaceae, especially in high-risk items like raw dairy products. The broad-spectrum infectivity exhibited by Phage_SH-2, Phage_OES_C-1, and Phage_Ra_O-2, along with their confirmed genomic safety, warrants their consideration for inclusion in phage cocktails. Future research should focus on testing their effectiveness in food-related environments, examining the dynamics of resistance development, and exploring regulatory approval processes to expedite their transition from research to practical use. The integrative approach used here, which merges traditional microbiological techniques with cutting-edge genomic and proteomic methods, provides a reproducible framework for the systematic discovery and validation of new therapeutic phages targeting antimicrobial-resistant pathogens.

It should be noted that the present study is primarily computational and genomic in scope. A key limitation is the lack of experimental validation of phage efficacy in food matrices or animal models. The biocontrol potential inferred from in vitro biological characterization and genomic safety profiling requires confirmation through in situ or in vivo challenge experiments before these phages can be considered for practical applications. Future studies should therefore prioritize testing phage cocktail performance directly in dairy and other food systems, as well as assessing stability under food-processing conditions.

## Data Availability

Data will be made available on request.

## Supplementary Materials

The following supporting information can be downloaded at: https://www.mdpi.com/article/10.3390/biology15070578/s1. File S1. Supplementary tables and figures.
