# Supplementary material for: Phage Frontiers: Genomic and Functional Profiling of Novel Virulent Agents Targeting Foodborne Enterobacteriaceae
Source: Biology (Basel). 2026 Apr 4;15(7):578. doi: 10.3390/biology15070578 (PMC13072308; doi:10.3390/biology15070578)
Supplement: Supplementary file 1 [file biology-15-00578-s001.zip › biology-4216956-supplementary.pdf]

SUPPLEMENTARY Materials

Phage Frontiers: Genomic and Functional Profiling of Novel Virulent Agents Targeting Foodborne Enterobacteriaceae

SUPPLEMENTARY Tables and Figures

Table S1. Summary of bacteriophage isolates, environmental sources, bacterial hosts, and GenBank accession numbers.

Source of samples	Bacterial Host	Phage Name	Accession Number
Waste water	*Escherichia coli* str. K-12 substr. MG1655	*Escherichia* phage OES_C-1	PQ553088
Sewage	*Escherichia coli* str. K-12 substr. MG1655	*Escherichia* phage OES_C-2	PQ642263
Waste water	*Escherichia coli* str. K-12 substr. MG1655	*Escherichia* phage OES_C-3	PQ642264
Waste water	*Shigella boydii* strain ESBL-W3-2	*Shigella boydii* phage SH-1	PQ642266
Sewage	*Shigella boydii* strain ESBL-W3-2	*Shigella boydii* phage SH-2	PQ642267
Waste water	*Raoultella ornithinolytica* strain YZSH173	*Raoultella ornithinolytica* phage Ra_O-1	PQ564427
Sewage	*Raoultella ornithinolytica* strain YZSH173	*Raoultella ornithinolytica* phage Ra_O-2	PQ642265
Waste water	*Obesumbacterium proteus* strain LE8	*Obesumbacterium proteus* phage Ob_P	PQ664488

Table S2. Morphological characteristics and plaque phenotypes of isolated bacteriophages.

Phage Isolates	Head diameter (nm)	Tail length (nm)	Shape of head	Family	Plaque Morphology
Phage OES_C-1	99	125	Icosahedral	*Myoviridae*	Clear without halo
Phage OES_C-2	101	95	Icosahedral	*Myoviridae*	Clear with halo
Phage OES_C-3	105	98.1	Icosahedral	*Myoviridae*	Clear without halo
Phage_SH-1	105	107	Icosahedral	*Myoviridae*	Clear without halo
Phage_SH-2	107	106	Icosahedral	*Myoviridae*	Clear with halo
Phage_Ra_O-1	118	114	Icosahedral	*Myoviridae*	Clear without halo
Phage_Ra_O-2	104	106	Icosahedral	*Myoviridae*	Clear with halo
Phage_Ob_ P	102	102	Icosahedral	*Myoviridae*	Clear without halo

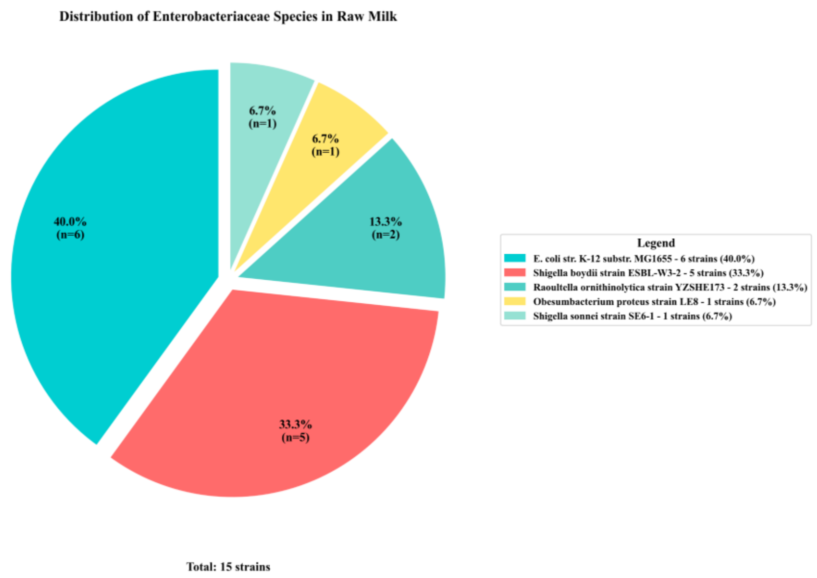

Figure S1. This analysis illustrates the distribution of various Enterobacteriaceae species identified in the raw milk samples examined in this study. *E. coli* str. K-12 substr. MG1655 was the most frequently detected species, identified in six strains, followed by *S. boydii* strain ESBL-M3-2, which was found in five strains. *R. ornithinolytica* strain YZSHE173 was present in two strains, whereas *O. proteus* strain LEB and *S. sonnei* strain SE6-1 were each detected in only one strain.

Table S3. Summary of gene prediction consensus rates across seven tools applied to eight bacteriophage genomes (moved from Section 3.7 of the main text per Reviewer 2 recommendation).

Phage	Total Genes	Consensus Genes	Consensus Rate (%)	Highest-Performing Tool
**Phage OES_C-1**	520	95	18.3	Decouphage (197 unique)
**Phage OES_C-2**	485	62	12.8	Decouphage (293 predictions)
**Phage OES_C-3**	492	68	13.8	Decouphage (225 unique)
**Phage_SH-1**	450	42	9.3	Decouphage (249 unique)
**Phage_SH-2**	468	52	11.1	Decouphage (268 unique)
**Phage_Ra_O-1**	485	48	9.9	Decouphage (267 unique)
**Phage_Ra_O-2**	478	44	9.2	Decouphage (268 unique)
**Phage_Ob_P**	230	25	10.9	Decouphage (165 unique)

Consensus defined as genes predicted by ⩾4 of 7 tools. Full per-phage UpSet plots and Jaccard concordance indices are provided in Figures S11–S18.

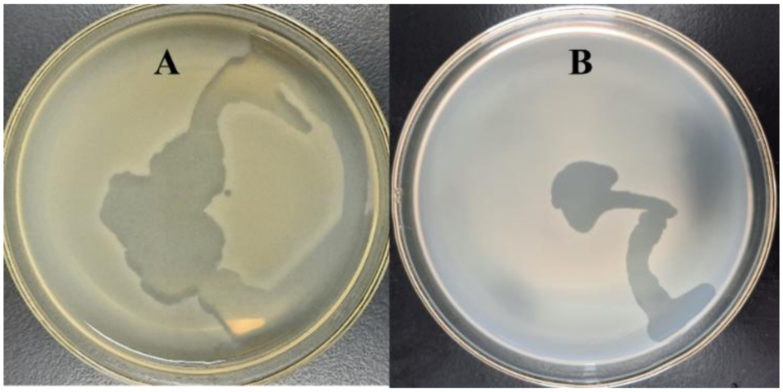

Figure S2. presents a spot test assay illustrating the activity of bacteriophages against Enterobacteriaceae strains isolated from environmental samples. This figure depicts the outcomes of spot tests performed on bacterial lawns using phage preparations derived from two distinct sample types: Sample A, representing wastewater, and Sample B, representing sewage. The clear zones of lysis (plaques) observed on the bacterial lawns indicate successful phage infection and replication, thereby demonstrating the lytic activity of the isolated bacteriophages against their bacterial hosts. Sample A was tested against multiple Enterobacteriaceae strains, including *E. coli*, *S. boydii*, *R. ornithinolytica*, and *O. proteus*. Sample B was evaluated against *E. coli*, *S. boydii*, and *R. ornithinolytica*, similarly demonstrating effective bacterial lysis.

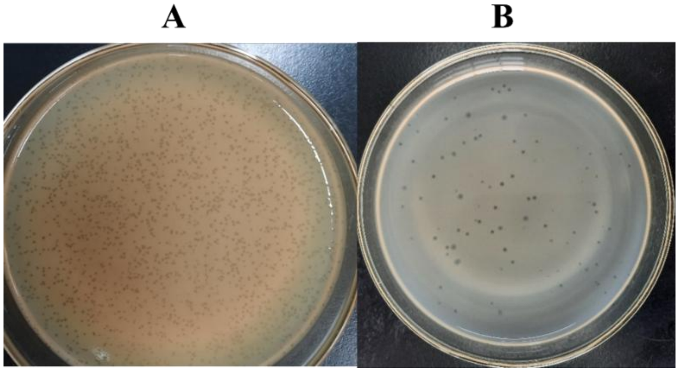

Figure S3. Morphological Variants in Plaque Assays. This figure illustrates two distinct plaque morphotypes identified during the characterization of bacteriophages: Type A (panel A), which lacks a halo, and Type B (panel B), which exhibits a prominent halo encircling the clear zones. These morphological distinctions are indicative of variations in phage-host interactions and enzymatic activity among the isolated phages. Type A plaques are defined by clear zones devoid of halos and are representative of Phage OES_C-1, Phage_SH-1, Phage_Ra_O-1, and Phage_Ob_P. These plaques manifest numerous small, uniform clear spots on the bacterial lawn, signifying efficient lytic activity but limited or absent depolymerase activity. The sharp boundaries of Type A plaques suggest that lysis is restricted to areas of direct phage infection without significant enzymatic degradation of surrounding bacterial structures. In contrast, Type B plaques display distinct halos around the clear lytic zones and are characteristic of Phage OES_C-2, Phage OES_C-3, Phage_SH-2, and Phage_Ra_O-2. The formation of halos indicates the production of polysaccharide depolymerases or other exoenzymes that degrade bacterial capsules or extracellular polysaccharides beyond the zone of direct bacterial lysis. These enzymes diffuse outward from the plaque center, creating translucent halo regions visible as lighter zones surrounding the completely cleared center.

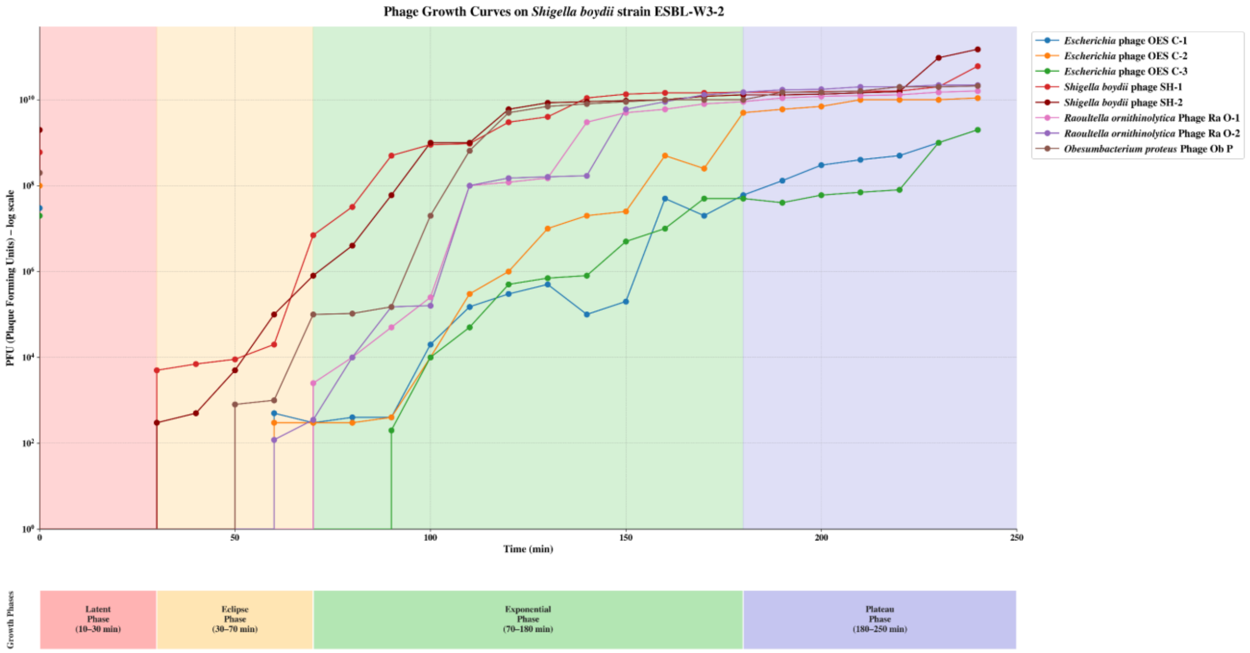

Figure S4. One-step growth curves of bacteriophages representing distinct morphotypes, analyzed using *S. boydii* as the host organism. The production analysis identified three distinct performance categories. The highest-producing phages were the native Shigella phages, Phage_SH-2 (1.50×10^{11} PFU, burst size: 74 particles/cell) and Phage_SH-1 (6.05×10^{10} PFU, burst size: 101 particles/cell), which demonstrated superior host adaptation with balanced burst sizes. A second category of proficient producers included Phage_Ra_O-2 (2.20×10^{10} PFU, burst size: 110 particles/cell), Phage_Ob_P (2.10×10^{10} PFU, burst size: 105 particles/cell), and Phage_Ra_O-1 (1.60×10^{10} PFU, burst size: 79 particles/cell), all of which exhibited strong cross-genus infectivity. The third category comprised moderate producers, namely Phage OES_C-2 (1.10×10^{10} PFU, burst size: 110 particles/cell), Phage OES_C-3 (2×10^{9} PFU, burst size: 99 particles/cell), and Phage OES_C-1 (2×10^{9} PFU, burst size: 66 particles/cell), which demonstrated reduced cross-genus efficiency. Notably, native *Shigella* phages dominated with the highest titers, while cross-genus phages exhibited relatively uniform burst sizes ranging from 66 to 110 particles per cell. Furthermore, analysis of host specificity revealed that native Shigella phages achieved titers 50-75-fold higher than *Escherichia* phages when propagated on the *Shigella* host, underscoring the significant advantage of host-phage compatibility.

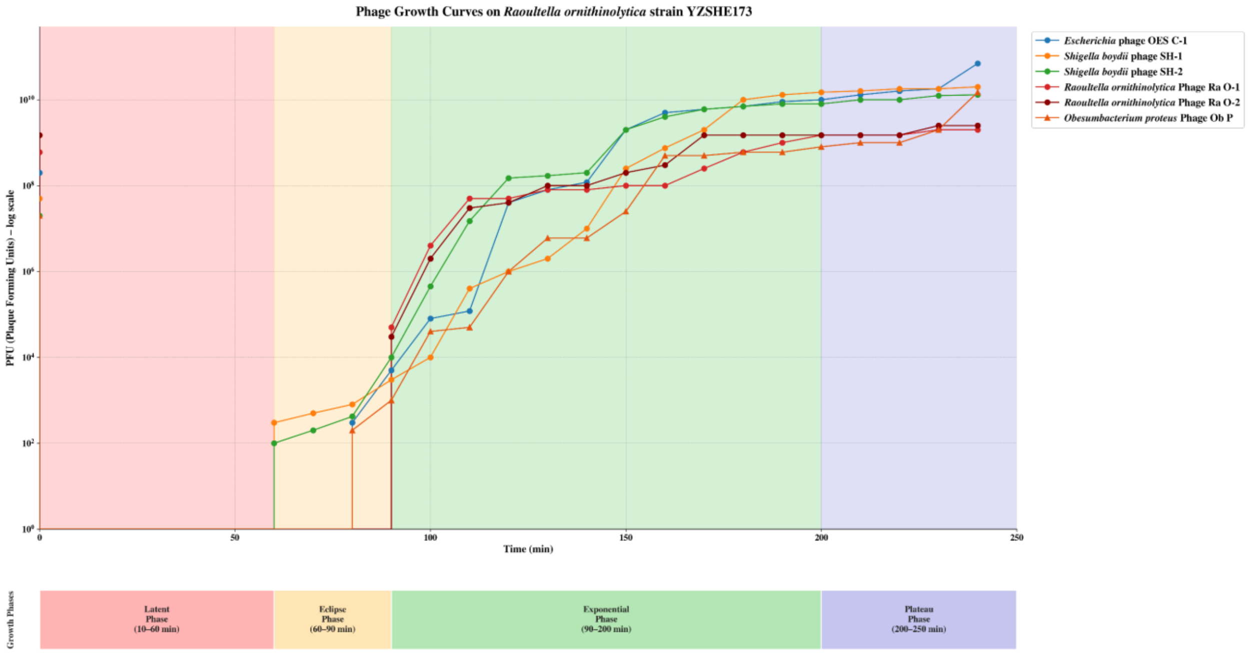

Figure S5. One-step growth curves of bacteriophages representing distinct morphotypes, analyzed using *R. ornithinolytica* as the host organism. The production analysis identified three distinct performance categories, revealing unexpected patterns of host-phage compatibility. The highest-producing phages were the cross-genus isolates Phage OES_C-1 (7×10^{10} PFU, burst size: 34 particles/cell) and Phage_SH-1 (2×10^{10} PFU, burst size: 39 particles/cell), with the Escherichia phage demonstrating exceptional cross-genus infectivity by achieving titers 35-fold higher than native *Raoultella* phages. A second category of proficient producers included Phage_SH-2 (1.30×10^{10} PFU, burst size: 64 particles/cell) and Phage_Ob_P (1.50×10^{10} PFU, burst size: 75 particles/cell), both exhibiting robust cross-genus adaptation with notably high burst sizes. Remarkably, the lowest producers were the native Phage_Ra_O-2 (2.50×10^{9} PFU, burst size: 16 particles/cell) and Phage_Ra_O-1 (2×10^{9} PFU, burst size: 33 particles/cell), which displayed unexpectedly poor performance on their cognate host strain. This analysis revealed a striking host specificity paradox, wherein non-native *Escherichia* phages dramatically outperformed native *Raoultella* phages by 28-35-fold, despite the latter being isolated from the same bacterial genus. The observed burst size range of 16-75 particles per cell, with characteristically lower burst sizes in native phages, suggests the potential involvement of host resistance mechanisms that may selectively limit native phage replication.

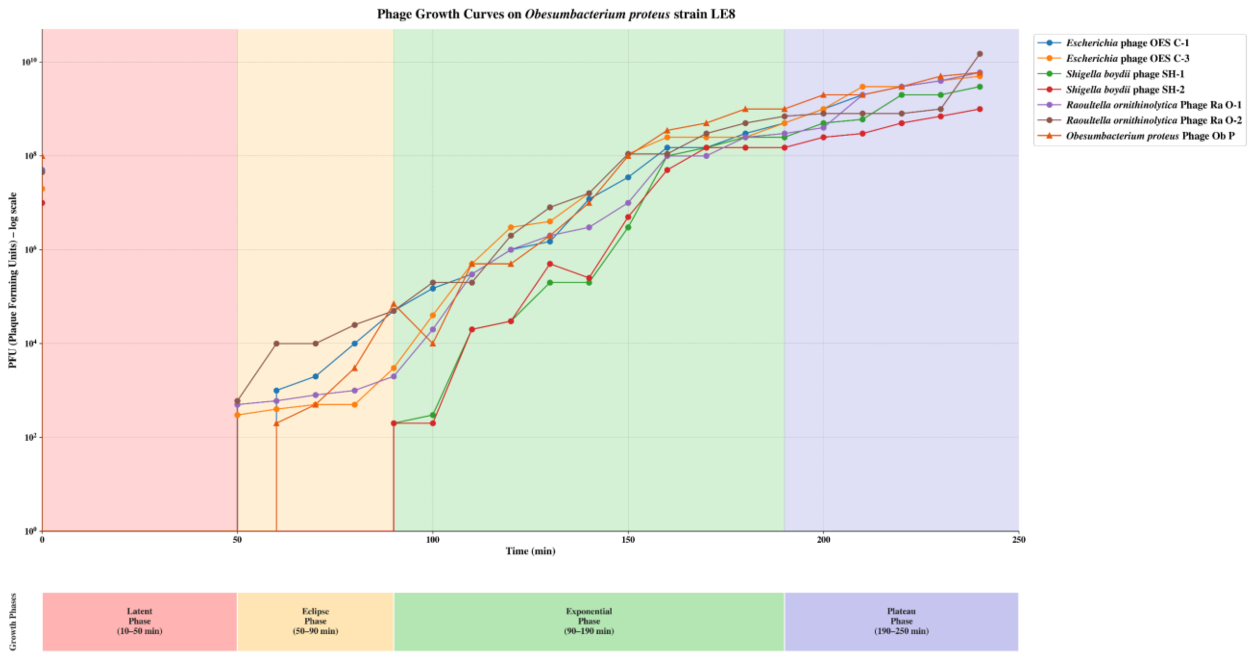

Figure S6. One-step growth curves of bacteriophages representing distinct morphotypes, analyzed using *O. proteus* as the host organism. The production analysis identified three distinct performance categories, with notable patterns of cross-genus compatibility. The highest-producing phages included cross-genus Phage_Ra_O-2 (1.50×10^{10} PFU, burst size: 133 particles/cell) and Phage_Ra_O-1 (6×10^{9} PFU, burst size: 127 particles/cell), Phage OES_C-1 (6×10^{9} PFU, burst size: 119 particles/cell), and the native Phage_Ob_P (6×10^{9} PFU, burst size: 59 particles/cell). Notably, *Raoultella* phages demonstrated exceptional cross-genus performance, dominating with the highest burst sizes ranging from 127 to 133 particles per cell. A second category of proficient producers included Phage OES_C-3 (5×10^{9} PFU, burst size: 25 particles/cell) and Phage_SH-1 (3×10^{9} PFU, burst size: 30 particles/cell), both exhibiting strong cross-genus infectivity despite substantially lower burst sizes. The moderate producer Phage_SH-2 (1×10^{9} PFU, burst size: 60 particles/cell) displayed balanced performance characteristics. A striking finding was that cross-genus *Raoultella* phages achieved titers 2.5-fold higher than the native *Obesumbacterium* phage while demonstrating superior burst efficiency. Furthermore, all tested phages successfully infected *Obesumbacterium*, revealing remarkable host versatility and broad host range across genera. The observed burst size range of 25-133 particles per cell highlighted the exceptional replication efficiency of *Raoultella* phages, which performed 2-5-fold higher than other phages tested, suggesting highly effective exploitation of the *Obesumbacterium* host machinery.

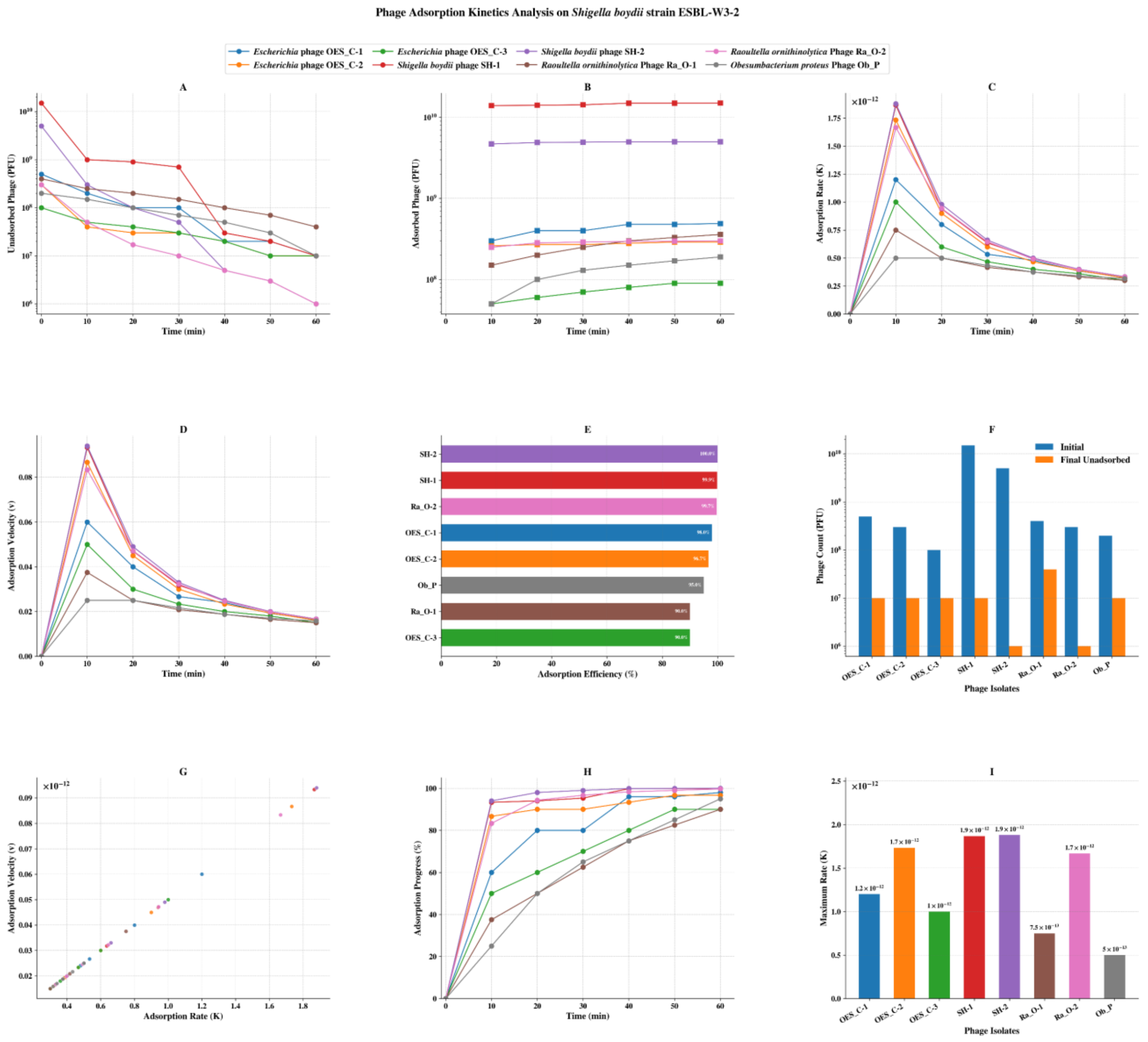

Figure S7. presents the adsorption kinetics of eight bacteriophage isolates, namely Phage OES_C-1, Phage OES_C-2, Phage OES_C-3, Phage_SH-1, Phage_SH-2, Phage_Ra_O-1, Phage_Ra_O-2, and Phage_Ob_P, on *S. boydii*. The temporal profiles illustrate (A) the decline of un-adsorbed phages, (B) the accumulation of adsorbed phages, (C) the adsorption rate (K), (D) the adsorption velocity (v), (E) the final adsorption efficiency, (F) a comparison between initial and final un-adsorbed phages, (G) the correlation between rate and velocity, (H) the normalized adsorption progress, and (I) a comparison of maximum adsorption rates. The homologous *Shigella* phages, Phage_SH-1 and Phage_SH-2, achieved near-complete adsorption efficiencies of 99.93% and 99.98%, respectively, with maximum rates of $1.87\text{-}1.88\times10^{-12}$ at 10 minutes, indicative of optimized receptor-binding through host-phage coevolution. The heterologous Phage_Ra_O-2 demonstrated significant cross-genus binding with an efficiency of 99.67% and a maximum rate of 1.67×10^{-12}, suggesting the conservation of surface receptor epitopes between the *Shigella* and *Raoultella* genera. *Escherichia* phages exhibited intermediate efficiencies ranging from 90% to 98%, reflecting their close phylogenetic relationships, while Phage_Ob_P achieved an efficiency of 95%. Strong correlations between rate and velocity ($R^2 > 0.93$) confirm the experimental reproducibility, with homologous *Shigella* phages achieving 94% binding within 10 minutes, compared to 20-30 minutes for heterologous phages.

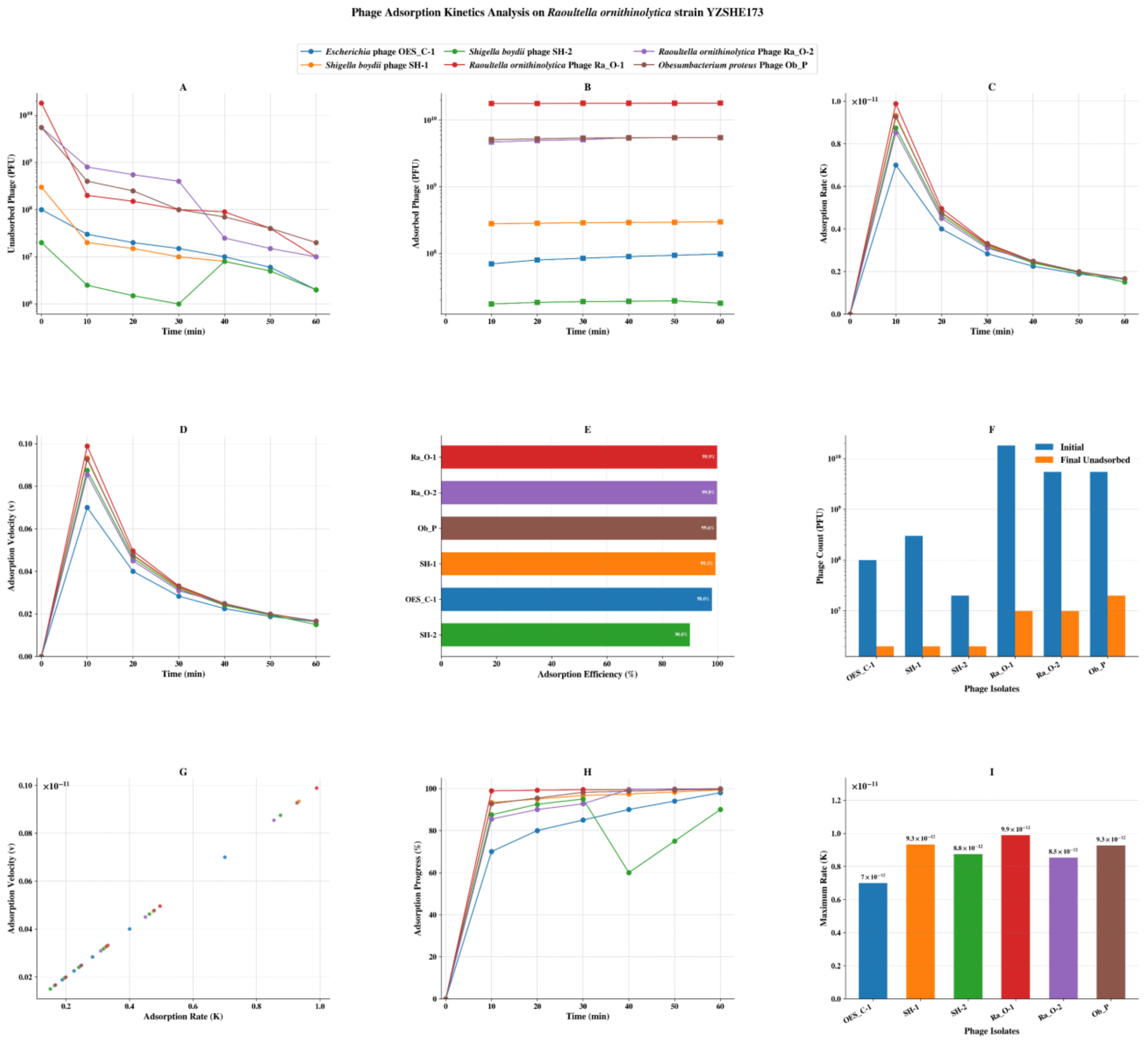

Figure S8. The adsorption kinetics of six bacteriophage isolates illustrates (Phage OES_C-1, Phage_SH-1, Phage_SH-2, Phage_Ra_O-1, Phage_Ra_O-2, and Phage_Ob_P) on *R. ornithinolytica*. The temporal profiles depict (A) the decline of un-adsorbed phages, (B) the accumulation of adsorbed phages, (C) the adsorption rate (K), (D) the adsorption velocity (ν), (E) the final adsorption efficiency, (F) a comparison between initial and final un-adsorbed phages, (G) the correlation between rate and velocity, (H) the normalized adsorption progress, and (I) a comparison of maximum adsorption rates. Homologous phages Phage_Ra_O-1 and Phage_Ra_O-2 exhibited exceptional binding efficiencies of 99.94% and 99.82%, respectively, with maximum adsorption rates of 9.89×10^{-12} and 8.55×10^{-12} at 10 minutes, indicative of optimized receptor recognition through host-phage coevolution. Notably, heterologous phages Phage_SH-1 and Phage_Ob_P demonstrated nearly equivalent performance, with efficiencies of 99.33% and 99.64% and maximum rates of 9.33×10^{-12} and 9.27×10^{-12}, respectively, suggesting significant conservation of surface receptor structures across Enterobacteriaceae genera. Phage OES_C-1 achieved an efficiency of 98% (maximum rate of 7×10^{-12}), while Phage_SH-2 exhibited 90% efficiency, representing the lowest yet substantial binding capacity among the tested isolates. Strong rate-velocity correlations ($R^2 > 0.95$) affirm experimental reproducibility, while normalized progress curves indicate that most phages achieved 90% binding within 10-20 minutes, underscoring the highly permissive nature of *R. ornithinolytica* surface receptors for diverse phage attachment.

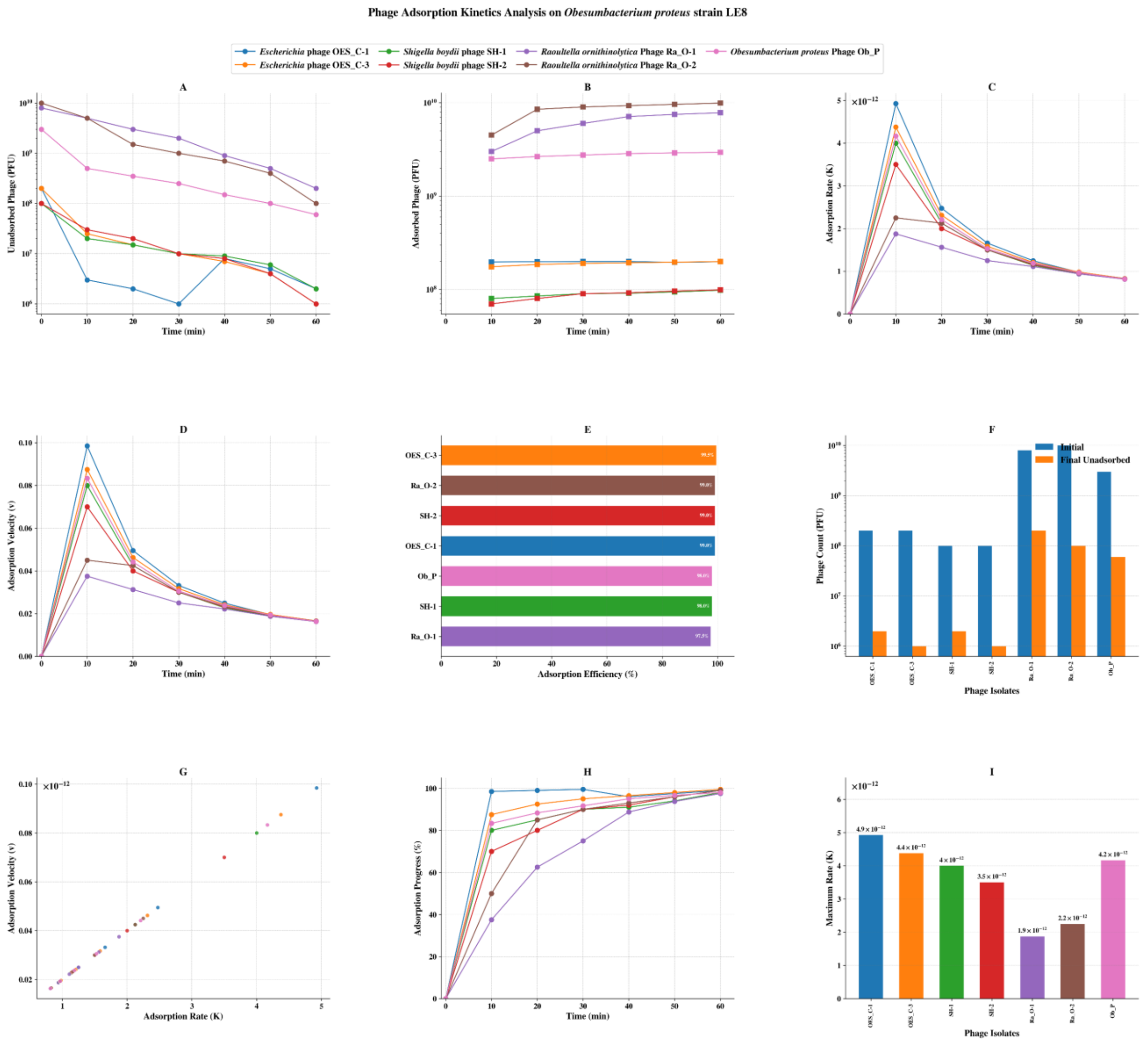

Figure S9. The adsorption kinetics of seven bacteriophage isolates illustrates (Phage OES_C-1, Phage OES_C-3, Phage_SH-1, Phage_SH-2, Phage_Ra_O-1, Phage_Ra_O-2, and Phage_Ob_P) on *O. proteus*. The temporal profiles depict (A) the decline of un-adsorbed phages, (B) the accumulation of adsorbed phages, (C) the adsorption rate (K), (D) the adsorption velocity (v), (E) the final adsorption efficiency, (F) a comparison of initial versus final un-adsorbed phages, (G) the correlation between rate and velocity, (H) the normalized adsorption progress, and (I) a comparison of maximum adsorption rates. Notably, heterologous Escherichia phages exhibited remarkable cross-genus attachment capabilities on *O. proteus*. Phage OES_C-1 and Phage OES_C-3 achieved binding efficiencies of 99% and 99.50%, with peak rates of 4.93×10^{-12} and 4.38×10^{-12}, respectively, closely mirroring the performance of the homologous Phage_Ob_P (98% efficiency, 4.17×10^{-12} peak rate). This notable similarity suggests significant receptor compatibility between the *Escherichia* and *Obesumbacterium* genera. *Shigella* phages also demonstrated strong cross-genus binding, with Phage_SH-1 and Phage_SH-2 achieving efficiencies of 98% and 99% at peak rates of 4×10^{-12} and 3.50×10^{-12}, respectively. *Raoultella* phages Phage_Ra_O-1 and Phage_Ra_O-2 exhibited slightly lower yet substantial binding, reaching efficiencies of 97.50% and 99% with rates of 1.88×10^{-12} and 2.25×10^{-12}. These findings collectively indicate that *O. proteus* presents broadly accessible surface receptors recognized by diverse Enterobacteriaceae phages. High rate-velocity correlations ($R^2 > 0.94$) confirm experimental consistency across all phages. Normalized kinetic analysis revealed that heterologous Escherichia and Shigella phages completed 70-87.5% of total binding within the first 10 minutes, aligning with the rapid attachment dynamics characteristic of cognate host-phage interactions. This kinetic similarity supports the functional conservation of receptor recognition machinery among phages infecting phylogenetically related Enterobacteriaceae genera.

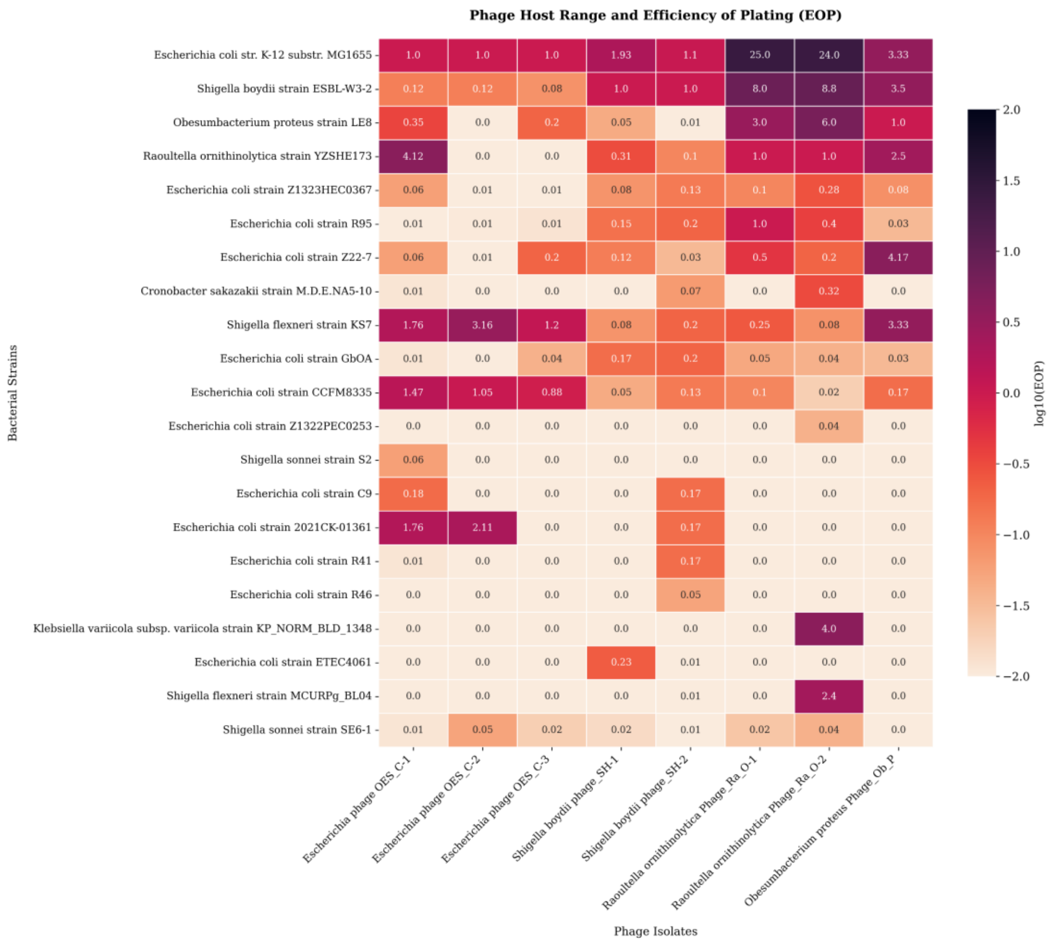

Figure S10. presents a heatmap illustrating the host range and efficiency of plating (EOP) of eight bacteriophage isolates across 21 bacterial strains from various Enterobacteriaceae genera. *E. coli* demonstrated the most permissive phenotype, with EOP values ranging from 1.0 to 25.0 for all phages, thereby establishing it as a universal propagation host. *S. boydii* exhibited broad susceptibility, with homologous phages achieving the highest efficiency (EOP 1.0) and heterologous phages showing reduced EOPs (0.08-1.93), indicative of shared receptor architectures between *Escherichia* and *Shigella*. *R. ornithinolytica* displayed a restricted host range, with high-efficiency infection only by the homologous Phage_Ra_O-1 (EOP 4.12), whereas *O. proteus* showed intermediate susceptibility (EOP 0.0-3.0). Most clinical isolates of *E. coli* exhibited highly restricted susceptibility (EOP 0.0-0.2), with notable exceptions such as strain 2021CK-01361, which showed high sensitivity to Phage OES_C-2 (EOP 2.11), suggesting strain-level variation in receptor expression and innate defense mechanisms. High-efficiency infections (EOP ≥1.0) are predominantly confined to homologous or phylogenetically related host-phage pairs, underscoring that receptor specificity and surface barriers impose significant host range restrictions.

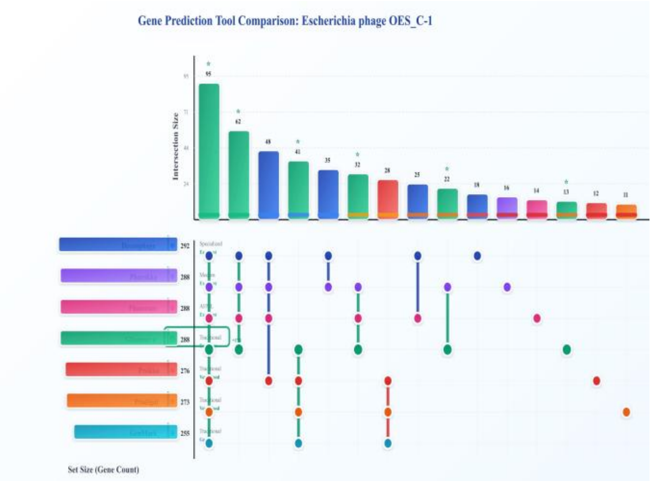

Figure S11. A comprehensive comparison of gene prediction tools for Phage OES_C-1, utilizing an UpSet plot visualization. The analysis identified a total of 520 unique genes across all prediction tools, with 95 genes predicted by all seven tools, representing an 18.3% universal consensus. The intersection sizes decrease progressively, with 62 genes in the second-largest intersection, followed by 48, 41, 35, 32, 28, 25, 22, 18, 16, 14, 13, 12, and 11 genes for various tool combinations. The highest-performing tool achieved 292 gene predictions (identified as Decouphage in the performance matrix), while Glimmer demonstrated exceptional performance with 288 predicted genes, significantly exceeding its typical range of 51-120 genes and representing a 17% improvement above baseline performance. The overall tool agreement reached 74.2%, indicating substantial concordance among prediction algorithms. Notable subsets include the "traditional tools harmony" of 41 genes and an "elite quartet" of 62 genes predicted with Glimmer. The performance matrix categorizes tools into three tiers: Excellent (≥285 genes: Decouphage, Phanotate, Pharokka, Glimmer), Very Good (≥270 genes: Prodigal, Prokka), and Good (≥260 genes: GeneMarkS), with OES_C-1 identified as Glimmer's ideal phage.

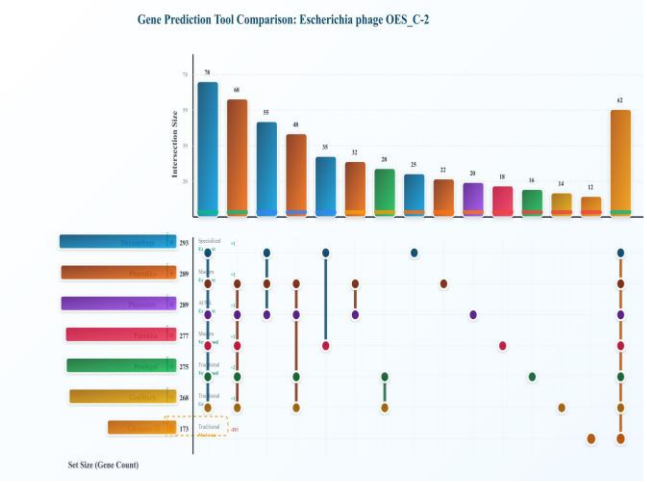

Figure S12. A comprehensive analysis of gene prediction tools for Phage OES_C-2, utilizing an UpSet plot visualization. This analysis identifies a total of 485 unique genes, with 62 genes predicted by all seven tools, indicating a significant universal consensus decrease of 33 genes compared to Phage OES_C-1, and constituting approximately 12.8% of the unique genes. The UpSet plot illustrates intersection sizes of 78, 68, 55, 48, 35, 32, 28, 25, 22, 20, 18, 16, 14, and 12 genes across decreasing tool combinations. Decouphage demonstrates the highest performance with 293 genes, an increase of one gene compared to Phage OES_C-1. Glimmer shows moderate improvement with 173 genes, a decrease of 115 genes compared to the typical Phage OES_C-1 range of 53-120, indicating moderate rather

than breakthrough performance and confirming sequence-dependent optimization patterns. The overall tool agreement of 66.8% is notably lower than that of Phage OES_C-1, suggesting that Phage OES_C-2 presents greater annotation challenges despite the increased universal consensus. The analysis identifies a 6-tool consensus of 78 genes as the optimal strategy, with sequence-specific insights indicating that Phage OES_C-1 was an exceptional case for Glimmer, while Phage OES_C-2 exhibits more typical performance patterns. The performance matrix categorizes tools into Excellent (≥285 genes: Decouphage, Pharokka, Phanotate), Very Good (≥270 genes: Prokka, Prodigal), Good (≥250 genes: GenMarkS), and Moderate (≥150 genes: Glimmer 173). Comparative performance indicates that most tools remain consistent with Phage OES_C-1, while Glimmer exhibits sequence sensitivity, thereby confirming that phage genome characteristics substantially influence the effectiveness of prediction tools.

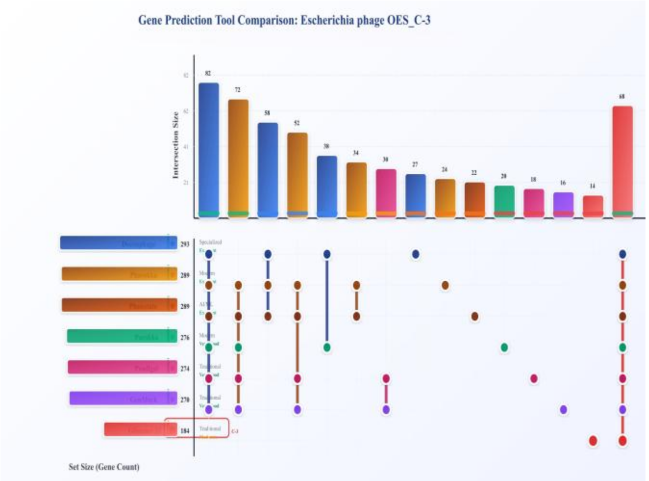

Figure S13. A comprehensive analysis of seven gene prediction tools applied to Phage OES_C-3, utilizing an UpSet plot visualization. This analysis identifies a total of 492 unique genes, with 68 genes consistently predicted by all seven tools, indicating an improvement in universal consensus (+6 compared to Phage OES_C-2) and accounting for approximately 13.8% of the unique genes. The UpSet plot illustrates intersection sizes of 82, 72, 58, 52, 38, 34, 30, 27, 24, 22, 20, 18, 16, and 14 genes across various tool combinations, with Decouphage demonstrating superior performance by predicting 293 genes. The Glimmer trilogy is now complete with 184 genes, reflecting a slight increase from the 173 genes identified in Phage OES_C-2, and the sequence-dependency pattern is consistently observed across the three-phage series (Phage OES_C-1: 288 exceptional breakthrough, Phage OES_C-2: 173 moderate improvement, Phage_OES_C-3: 184 slight uptick). This pattern confirms that phage-specific genome characteristics significantly influence Glimmer's effectiveness. The overall tool agreement reaches 68.5%, marking an improvement over Phage OES_C-2 (66.8%) and nearing the levels observed in Phage OES_C-1, suggesting that Phage OES_C-3 offers more favorable conditions for annotation. The performance matrix categorizes the tools into Excellent (≥285 genes: Decouphage, Pharokka, Phanotate), Very Good (≥270 genes: Prokka, Prodigal, GenMark), and Moderate (≥150 genes: Glimmer variable). The research conclusions emphasize that tool-phage compatibility varies significantly, precision annotation necessitates a confirmed 6-tool consensus strategy, and the 6-tool consensus remains optimal across the trilogy. This demonstrates that bacteriophage genome annotation requires adaptable, multi-tool approaches tailored to sequence-specific characteristics.

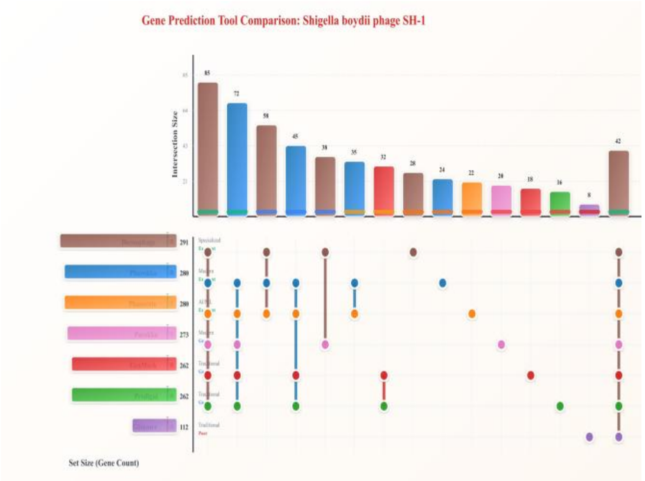

Figure S14. A comprehensive analysis of seven gene prediction tools applied to Phage_SH-1, utilizing an UpSet plot visualization. This analysis identifies a total of 450 unique genes, with 42 genes achieving universal consensus (9.3%), indicating a relatively modest level of agreement among all seven prediction tools. The UpSet plot illustrates intersection sizes of 85, 72, 58, 45, 38, 35, 32, 28, 24, 22, 20, 18, 16, and 8 genes across decreasing combinations of tools. Decouphage demonstrates peak performance, identifying 291 genes and establishing the baseline for the *S. boydii* series. The high-performance cluster of six tools identifies 85 genes, while modern tools show synergy with 72 genes. Phage-specialized pairings contribute 38 genes, and traditional tools demonstrate a benefit from Glimmer exclusion, with tool-specific discoveries totaling 138 genes. Collectively, these findings suggest that Phage_SH-1 presents specific challenges that necessitate diverse algorithmic approaches. The average tool agreement is 62.8%, the lowest among comparable phages analyzed, indicating that Phage_SH-1 presents particularly complex annotation challenges. Notably, Glimmer encounters substantial difficulties, identifying only 112 genes (compared to 288 in Phage_OES_C-1), representing a significant 176-gene deficit and classifying it as Poor (under 200). This highlights significant SH-1-specific challenges for this prediction tool. The performance matrix categorizes tools into Excellent (≥280 genes: Decouphage, Pharokka, Phanotate), Good (260–279: Prokka, GenMark, Prodigal), and Poor (under 200: Glimmer 112). Comparative insights reveal Jaccard similarities of 0.312–0.845 across tool pairs, recommending the exclusion of Glimmer for optimal Phage_SH-1 annotation. These findings underscore that bacteriophage genome characteristics can significantly influence individual tool performance, necessitating careful tool selection based on genome-specific validation.

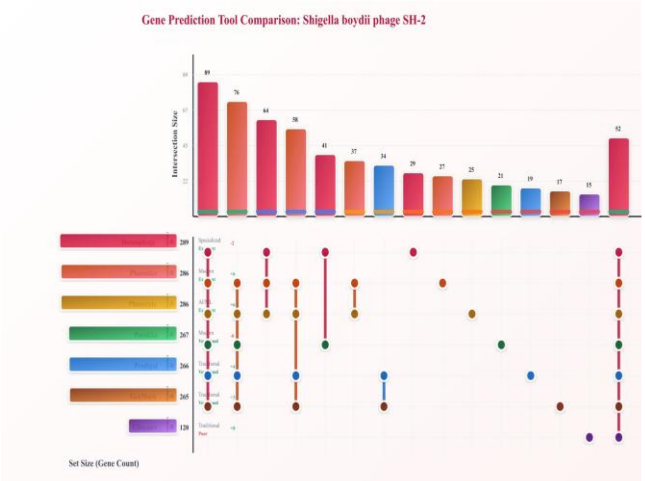

Figure S15. A comprehensive analysis of seven gene prediction tools applied to Phage_SH-2, utilizing an UpSet plot visualization. This analysis identifies a total of 468 unique genes across all tools, with 52 genes representing a universal consensus, an increase of 10 genes compared to Phage_SH-1, accounting for approximately 11.1% of the unique genes. The UpSet plot illustrates decreasing intersection sizes, starting with 89, 76, 64, 58, 41, 37, 34, 29, 27, 25, 21, 19, 17, and 15 genes across various tool combinations. Decouphage demonstrates the highest performance

with 289 genes, a decrease of 2 genes compared to Phage_SH-1. Notably, Glimmer exhibits significant improvement, identifying 120 genes, an increase of 8 genes relative to Phage_SH-1, thereby surpassing its typical performance range of 51-120 genes and indicating enhanced phage-specific optimization for the Phage_SH-2 variant. The analysis highlights high performance in a 6-tool cluster, yielding 89 genes, an increase of 4 genes compared to Phage_SH-1. Pharokka and Phanotate each contribute an additional 6 genes, while GenMark and Prodigal add 3 and 4 genes, respectively, collectively demonstrating superior overall performance and more prediction-friendly characteristics compared to Phage_SH-1. The average tool agreement of 65.2% suggests moderate but improved concordance. The performance matrix categorizes tools into Excellent (≥285 genes: Decouphage, Pharokka, Phanotate), Very Good (≥270 genes: Prokka, Prodigal), Good (≥260 genes: GeneMarkS), and Moderate (≥150 genes: Glimmer 120), indicating that Phage_SH-2 is more amenable than Phage_SH-1, with a 6-tool consensus remaining optimal and all-tool consensus demonstrating optimal base-level agreement.

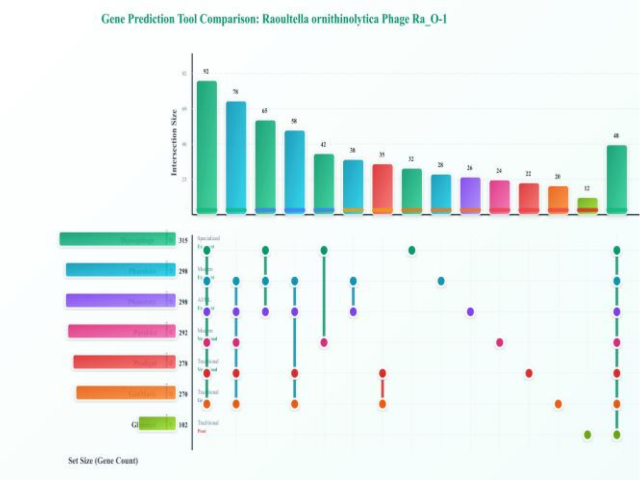

Figure S16. A comprehensive analysis of seven gene prediction tools applied to Phage_Ra_O-1, utilizing an UpSet plot visualization. This analysis identifies a total of 485 unique genes, with 48 genes achieving universal consensus (9.9%), indicating a relatively modest level of agreement among all seven tools. The UpSet plot illustrates intersection sizes of 92, 78, 65, 58, 42, 38, 35, 32, 28, 26, 24, 22, 20, and 12 genes across decreasing combinations of tools. Notably, Decouphage demonstrates exceptional peak performance, identifying 315 genes, which is 24 more than the maximum identified for Phage_SH-1, thereby underscoring its superior effectiveness for this specific phage genome. The modern trio of tools identifies 65 genes, while traditional tools show improvement with 35 genes. Decouphage maintains a substantial advantage of 32 unique genes over other high-performing tools, collectively suggesting Phage_Ra_O-1-specific advantages in genome architecture that favor certain prediction algorithms. The average tool agreement is 68.4%, indicating strong overall concordance despite the relatively low universal consensus percentage. However, Glimmer consistently underperforms, identifying only 102 genes (classified as Poor, under 200), which is substantially below its potential range, suggesting that Phage_Ra_O-1 presents specific challenges for this tool. The performance matrix categorizes tools into Excellent (≥295 genes: Decouphage, Pharokka, Phanotate), Very Good (≥275 genes: Prokka, Prodigal), Good (≥250 genes: GenMark), and Poor (under 200: Glimmer 102). This analysis emphasizes the identification of Phage_Ra_O-1-specific advantages, the establishment of an optimal 6-tool consensus strategy, and the consistent underperformance of Glimmer across this genome, highlighting the critical importance of employing multiple complementary tools for comprehensive bacteriophage genome annotation.

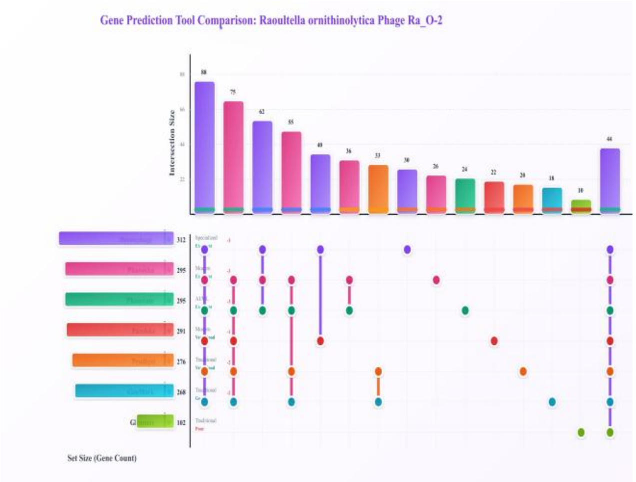

Figure S17. A comprehensive analysis of seven gene prediction tools applied to Phage_Ra_O-2, utilizing an UpSet plot visualization. This analysis identifies a total of 478 unique genes, with 44 genes achieving universal consensus, representing approximately 9.2% agreement among all seven tools. The UpSet plot illustrates intersection sizes of 88, 75, 62, 55, 40, 36, 33, 30, 26, 24, 22, 20, 18, and 10 genes across various tool combinations. Decouphage demonstrates peak performance with 312 genes, indicating consistent high-level effectiveness across both *Raoultella* phage genomes. The high-performance cluster of six tools identifies 88 genes, while the elite phage trio contributes 62 genes, and traditional tools yield 33 genes, collectively indicating consistent genus-level performance patterns with slight decreases across all categories. The average tool agreement is 66.8%, a modest decrease from Phage_Ra_O-1's 68.4%, yet maintaining substantial overall concordance. Glimmer continues to exhibit poor performance with 102 genes, reinforcing its persistent challenges with *Raoultella* phage genomes. The performance matrix categorizes tools into Excellent (≥295 genes: Decouphage, Pharokka, Phanotate), Very Good (≥275 genes: Prokka, Prodigal), Good (≥250 genes: GenMark), and Poor (under 200: Glimmer 102). Comparative patterns reveal small decreases versus Phage_Ra_O-1, consistent *Raoultella* genus-level performance maintenance, similar tool ranking preservation, and genus-consistent patterns, emphasizing that bacteriophage genome annotation exhibits both strain-specific variations and genus-level consistencies that must be considered when selecting optimal prediction strategies.

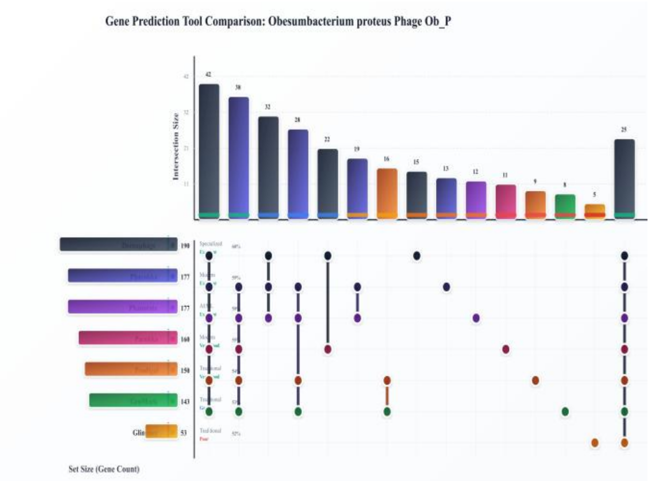

Figure S18. A comprehensive evaluation of seven gene prediction tools applied to the compact Phage_Ob_P genome, utilizing an UpSet plot for visualization. This analysis reveals a significantly smaller genome, comprising only 230 unique genes and 25 universal consensus genes (10.9%), which represents a 60% reduction compared to larger phages previously analyzed. The UpSet plot illustrates intersection sizes of 42, 38, 32, 28, 22, 19, 16, 15, 13, 12, 11, 9, 8, and 5 genes across various tool combinations. Notably, Decouphage demonstrates superior performance, identifying 190 genes, thereby maintaining its status as the highest-performing tool despite the reduced genome scale. The hierarchy

of tool performance is preserved at this smaller scale, with leading tools maintaining proportional gene counts, including Glimmer (177 genes), Phanotate and Pharokka (177 each), Prokka (160), Prodigal (150), and GenMark (143). In contrast, Glimmer exhibits a significant limitation, identifying only 53 genes (52% performance), a severe limitation compared to its typical range. Insights from the compact genome reveal a high-performance cluster of six tools identifying 42 genes and an elite phage trio identifying 32 genes. Despite the 60% reduction from larger phages, relative proportions are maintained and tool rankings remain consistent across scales, indicating that smaller bacterial genomes uniformly challenge all prediction tools. The scale comparison performance matrix indicates excellent performance by Decouphage (60% of larger phage predictions), very good performance by Pharokka/Phanotate (59% each), good performance by Prokka (55%) and Prodigal (54%), and poor performance by Glimmer (52%, a severe limitation). This emphasizes that small genomes present universal challenges to all annotation tools while maintaining predictable relative performance hierarchies.

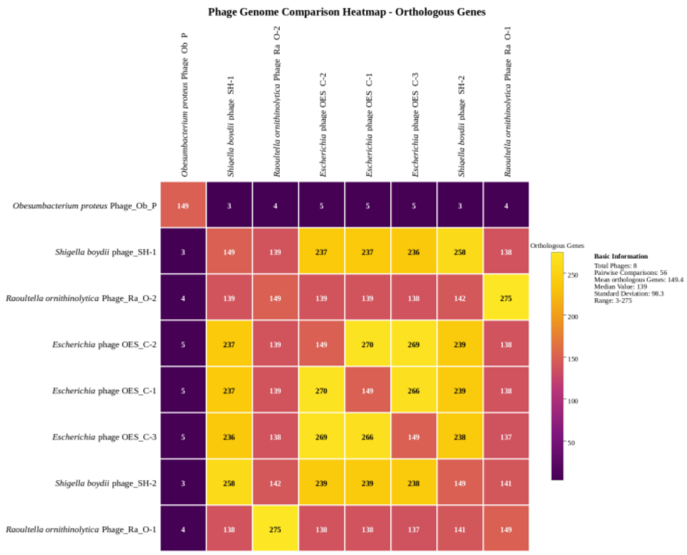

Figure S19. A heatmap depicting the absolute number of orthologous genes shared between each pair of eight bacteriophage genomes, offering a quantitative evaluation of conserved genetic content. The diagonal values indicate the total gene count for each phage genome, ranging from 149 genes (Phage_Ob_P) to 277 genes (Phage_Ra_O-1). The analysis demonstrates moderate variation in genome size among these phages, with *Escherichia* phages containing 273-274 genes, *Shigella* phages harboring 262-265 genes, *Raoultella* phages possessing 276-277 genes, and *Obesumbacterium* phage containing 149 genes, indicating that Phage_Ob_P has a substantially smaller genome, approximately half the size of the others. The highly related phage pairs exhibit remarkable absolute gene conservation: Phage OES_C-1 and Phage OES_C-2 share 270 orthologous genes out of their 273-274 gene repertoires, representing near-complete genome conservation with only 3-4 unique genes per phage; Phage_SH-1 and Phage_SH-2 share 258 of their 262-265 genes; and Phage_Ra_O-1 and Phage_Ra_O-2 share an impressive 275 of their 276-277 genes, leaving only 1-2 unique genes per genome, likely representing recently acquired accessory genes, minor sequence variations, or annotation differences. Cross-genus ortholog counts between *Escherichia* and *Shigella* phages range from 236-239 genes, representing approximately 86-91% of the smaller genome, indicating these phages retain the vast majority of their genetic toolkit across genera, with differences likely confined to tail fiber genes (determining host specificity), minor structural proteins, or regulatory elements. *Raoultella* phages share 137-142 orthologous genes with *Escherichia* and *Shigella* phages, representing approximately 50% overlap, suggesting the conserved core includes essential functions such as DNA replication machinery, major capsid and tail proteins, DNA packaging components, and fundamental lysis genes, while genus-specific genes likely encode unique host recognition systems, DNA modification enzymes, and auxiliary metabolic genes adapted to their respective hosts. Phage_Ob_P shares merely 3-5 orthologous genes with other phages, representing only 2-3% of its 149-gene genome. These few conserved genes likely represent only the most ancient and universal phage genes, such as highly conserved DNA polymerase catalytic domains or major capsid protein structural motifs. This minimal overlap, combined with its smaller genome size, suggests Phage_Ob_P may represent a different viral order or family, possibly with a fundamentally different genome organization, replication strategy, or structural architecture.

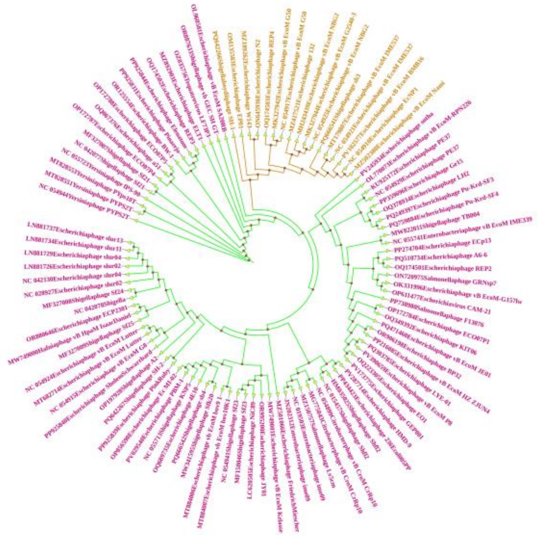

Figure S20. A circular phylogenetic tree delineating the evolutionary relationships of Phage_SH-1 based on the amino acid sequences of the terminase large subunit (TerL). The tree employs color-coded branches to differentiate various evolutionary lineages. Notably, the orange and yellow branches emphasize the clade where Phage_SH-1 clusters with its closest relatives, predominantly phages that infect *Escherichia* and *Shigella* species. This group constitutes a compact monophyletic cluster, characterized by short branch lengths indicative of recent divergence from a common ancestor and significant sequence similarity. The branch lengths reflect the extent of evolutionary divergence, with Phage_SH-1 positioned within a well-supported clade of *Escherichia*/*Shigella* phages near the top of the tree.

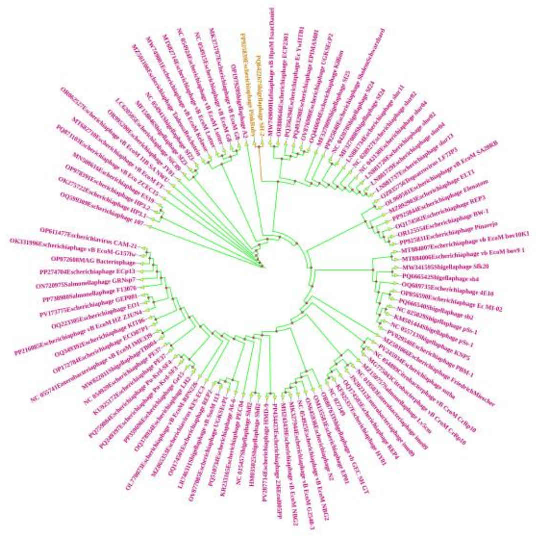

Figure S21. The circular phylogenetic tree illustrates the evolutionary relationships of Phage_SH-2 based on terminase large subunit (TerL) amino acid sequences. Color-coded branches throughout the tree represent different evolutionary lineages. The orange and yellow branches highlight the clade where Phage_SH-2 groups with its nearest relatives, primarily phages that infect *Escherichia* and *Shigella* species, which together form a compact monophyletic cluster. Short branch lengths within this clade reflect recent common ancestry and high levels of sequence similarity among these closely related phages.

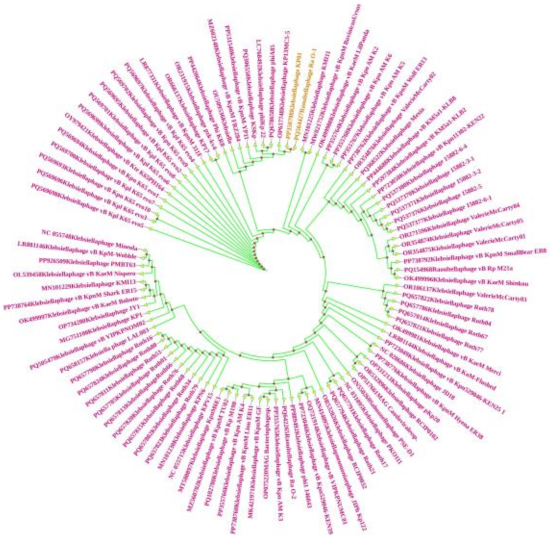

Figure S22. A circular phylogenetic tree delineating the evolutionary relationships of Phage_Ra_O-1 based on the amino acid sequences of the terminase large subunit (TerL). The branches of the tree are color-coded to indicate distinct phylogenetic lineages. Notably, the orange and yellow branches highlight the clade where Phage_Ra_O-1 clusters with its closest relatives, predominantly *Klebsiella* phages, underscoring the close taxonomic association between *Raoultella* and *Klebsiella* bacterial hosts. The relatively short branch lengths within this clade suggest a recent common ancestry and significant sequence similarity among these phages.

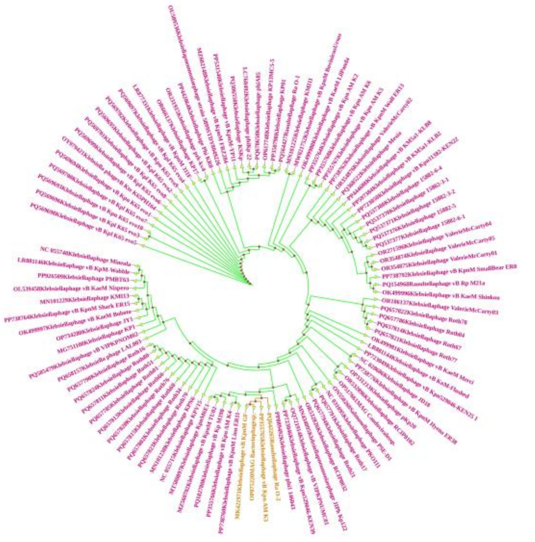

Figure S23. A phylogenetic analysis of Phage_Ra_O-2 utilizing terminase large subunit (TerL) amino acid sequences. The circular phylogenetic tree delineates the evolutionary relationships based on TerL amino acid sequences, with branches color-coded to represent distinct evolutionary lineages. The orange and yellow branches specifically highlight the clade in which Phage_Ra_O-2 is grouped with its closest relatives, predominantly *Klebsiella* phages. This clustering indicates a close phylogenetic relationship between *Raoultella* and *Klebsiella* bacterial hosts. The short branch lengths within this clade suggest recent divergence from a common ancestor and significant sequence similarity among these phages. The phylogenetic structure demonstrates that Phage_Ra_O-2 constitutes a distinct lineage of phages that specifically target the *Klebsiella*-*Raoultella* bacterial complex, exhibiting evolutionary differentiation from other Enterobacteriaceae phage groups.

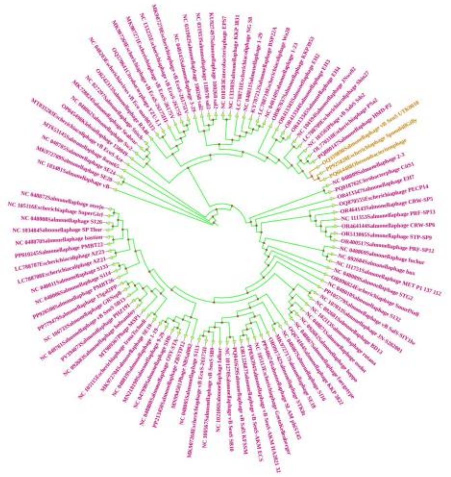

Figure S24. A circular phylogenetic tree elucidating the evolutionary relationships of Phage_Ob_P, utilizing terminase large subunit (TerL) amino acid sequences. The branches of the tree are color-coded to indicate distinct phylogenetic lineages. Notably, the orange and yellow branches highlight the clade where Phage_Ob_P clusters with its closest relatives, which include phages that infect *Salmonella* and related genera within the Enterobacteriaceae family. This positioning emphasizes the phylogenetic distinctiveness of Phage_Ob_P in comparison to the other phages analyzed in this study. The phylogenetic structure indicates that Phage_Ob_P constitutes a unique lineage, exhibiting significant evolutionary divergence from the other phages isolated in this investigation.

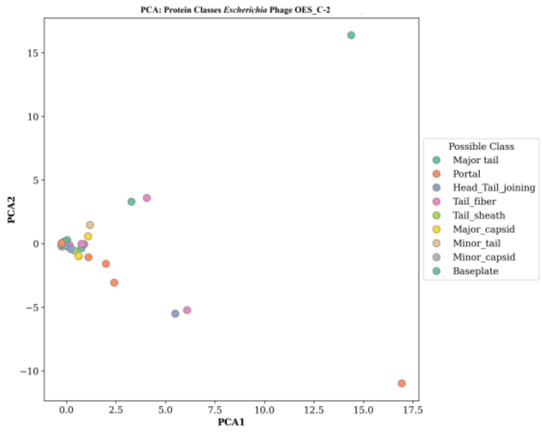

Figure S25. Principal Component Analysis (PCA) of the structural proteins of Phage OES_C-2 reveals distinct functional clustering patterns. Portal proteins exhibit notable dispersion, with variants distributed across the plot, suggesting specialized DNA packaging domains or unique amino acid compositions. Major tail proteins are positioned at extreme points, indicating highly divergent biochemical properties, likely due to extended coiled-coil domains and multimeric assembly requirements for tail tube structures. Tail fiber proteins display moderate dispersion in two groups: one near the origin and another at mid-range, implying conserved structural domains alongside variable receptor-binding regions. Head-tail joining proteins cluster centrally, reflecting similar biochemical properties necessary for head-tail linkage. The major capsid protein is positioned near the origin, indicating high conservation of the primary scaffolding protein forming the icosahedral head. Minor tail and capsid proteins cluster tightly near the origin, demonstrating conserved properties among accessory structural components. Tail sheath proteins show minimal dispersion, reflecting the conserved requirements of the contractile mechanism. Baseplate proteins cluster centrally, sharing characteristics with core structural components essential for host recognition.

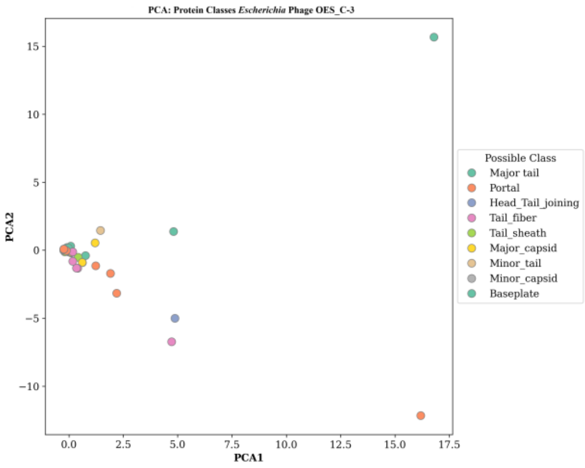

Figure S26. Principal Component Analysis (PCA) of Phage OES_C-3 structural proteins reveals distinct functional clustering patterns. Major tail proteins exhibit significant dispersion, indicating highly specialized biochemical characteristics pertinent to tail tube assembly and multimeric structure formation. Portal proteins demonstrate wide dispersion with multiple variants, reflecting biochemical diversity across the DNA packaging motor, genome translocation, and head-tail connector domains. Tail fiber proteins display moderate variability, balancing conserved structural frameworks with variable receptor-binding domains associated with host specificity. Head-tail joining proteins cluster centrally, exhibiting conserved properties essential for capsid-tail attachment. The major capsid protein clusters tightly near the origin, reflecting the high conservation required for stable icosahedral geometry and resistance to internal DNA pressure. Minor structural proteins (tail, capsid, and sheath) cluster near the origin with conserved biochemical properties, indicating strong evolutionary constraints necessary for coordinated assembly and mechanical function.

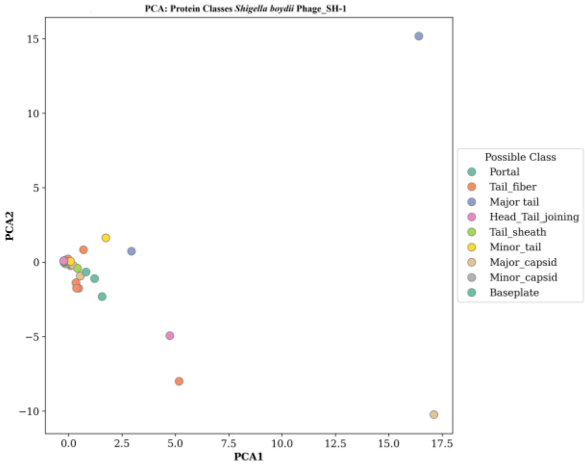

Figure S27. Principal Component Analysis (PCA) of Phage_SH-1 structural proteins elucidates functional modularity through distinct clustering patterns. Major tail proteins exhibit significant dispersion, indicative of biochemical heterogeneity that likely reflects a modular tail architecture with specialized variants. Tail fiber proteins demonstrate variable receptor-binding adaptations while maintaining conserved structural frameworks. Portal, head–tail joining, and minor structural proteins (tail, capsid, sheath, and baseplate) cluster near the origin, exhibiting conserved biochemical properties essential for assembly and structural stability. Notably, the major capsid protein occupies an atypical extreme position, suggesting unique biochemical characteristics potentially associated with stability adaptations. This distribution illustrates evolutionary constraints that favor variability in host-interacting components (tail proteins) while preserving core structural features.

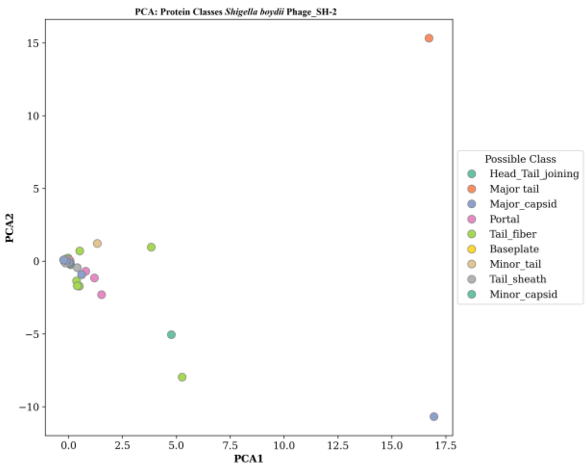

Figure S28. Principal Component Analysis (PCA) of the structural proteins of Phage_SH-2 reveals distinct functional clustering patterns. Major tail proteins are positioned at extreme points, indicating highly specialized biochemical characteristics associated with tail tube structure, assembly coordination, and contraction mechanics during DNA injection. Tail fiber proteins exhibit notable dispersion, characterized by conserved structural domains and variable receptor-binding regions involved in *Shigella* surface recognition. Head–tail joining proteins cluster centrally, displaying conserved connector properties essential for head-tail bridging. The major capsid protein occupies an unusual extreme lower-right position, suggesting distinctive biochemical characteristics potentially related to capsid assembly adaptations, stability requirements, or interactions with the DNA packaging machinery. Portal, baseplate, minor tail, tail sheath, and minor capsid proteins cluster tightly near the origin, exhibiting highly conserved properties required for coordinated assembly and mechanical function. This pattern demonstrates functional modularity, with specialized tail components exhibiting greater diversity, while core structural proteins remain conserved.

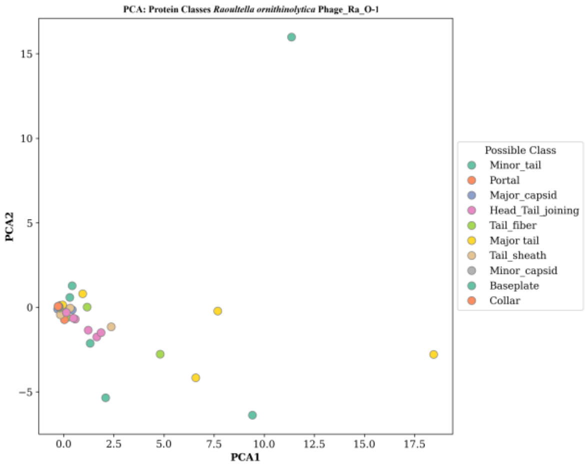

Figure S29. Principal Component Analysis (PCA) of Phage_Ra_O-1 structural proteins reveals distinct clustering patterns indicative of functional diversity. Minor tail proteins exhibit significant dispersion across the plot, suggesting substantial biochemical heterogeneity with multiple variants, potentially reflecting a complex tail architecture adapted for *Raoultella* cell surface attachment and specialized infection mechanisms. Major tail proteins also display wide dispersion with extreme positioning, indicating varied biochemical characteristics associated with distinct structural domains or functional specializations. Tail fiber proteins demonstrate moderate clustering, maintaining conserved structural features alongside variable host-recognition domains adapted to *Raoultella* surface receptors. Baseplate proteins show considerable dispersion, indicating biochemical diversity among components coordinating infection. Collar proteins differentiate Phage_Ra_O-1 from *Escherichia* and *Shigella* phages, suggesting architectural differences in the head–tail connection. Major capsid, head-tail joining, tail sheath, minor capsid, and portal proteins cluster near the origin, exhibiting conserved biochemical properties. This pattern illustrates functional modularity,

with greater diversity in tail-associated proteins adapted to *R. ornithinolytica*, while core capsid components remain conserved, reflecting host specialization during phage lineage evolution.

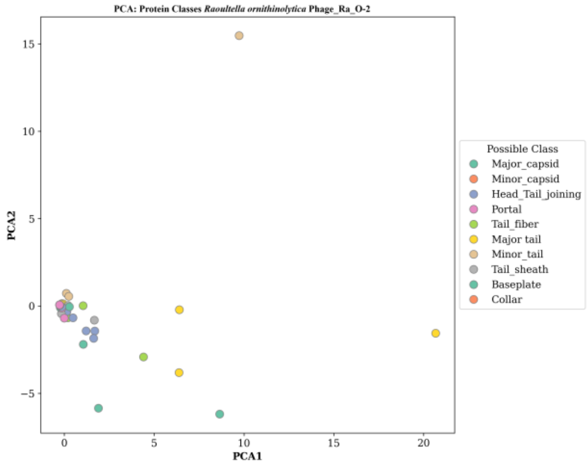

Figure S30. Principal Component Analysis (PCA) of the structural proteins of Phage_Ra_O-2 reveals distinct clustering patterns indicative of functional diversity. Minor tail proteins exhibit extreme positioning, suggesting highly specialized biochemical characteristics potentially associated with baseplate assembly, tail tip structures involved in initial host contact, or unique mechanical properties pertinent to *Raoultella* infection. Major tail proteins demonstrate notable dispersion, implying the presence of multiple variants with distinct structural properties optimized for different tail regions or assembly stages. Tail fiber proteins exhibit moderate clustering, maintaining conserved structural frameworks alongside diversified host-recognition domains adapted to *Raoultella* surface receptors. Baseplate proteins show considerable dispersion, indicating biochemical diversity among components that coordinate host recognition, tail contraction, and DNA injection. Collar proteins differentiate Phage_Ra_O-2 from *Escherichia* and *Shigella* phages, suggesting architectural differences. Major capsid, minor capsid, head-tail joining, portal, and tail sheath proteins cluster near the origin, reflecting conserved biochemical properties.

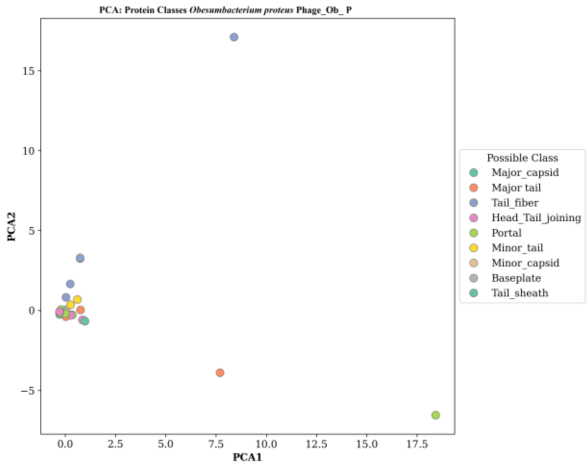

Figure S31. Principal Component Analysis (PCA) of Phage_Ob_P structural proteins reveals distinct clustering patterns indicative of functional diversity. Tail fiber proteins exhibit the most pronounced dispersion across the plot, suggesting significant biochemical heterogeneity that likely reflects the complex host-recognition requirements of *O. proteus*, a rare and poorly characterized host. The extreme positioning of tail fibers implies unique amino acid compositions, distinctive structural motifs, or specialized domains adapted for recognizing *Obesumbacterium* surface structures. Major tail proteins are positioned in isolated regions, indicating distinctive biochemical characteristics.

Portal proteins also occupy extreme positions, suggesting unique properties of the DNA packaging machinery. Major capsid, head-tail joining, minor tail, minor capsid, baseplate, and tail sheath proteins cluster closely near the origin, exhibiting conserved biochemical properties despite substantial genomic divergence (39-45% AAI), indicating strong structural constraints across phylogenetically distant phages.

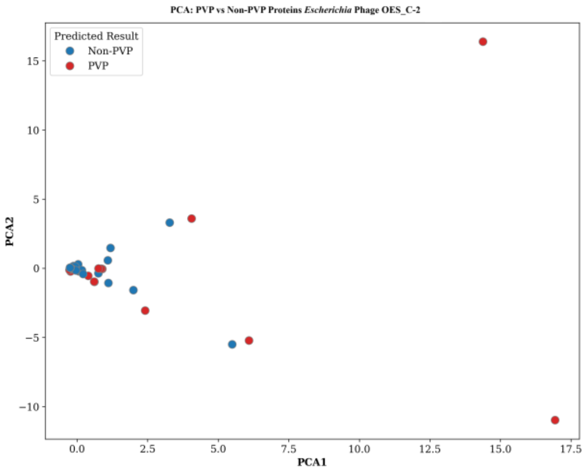

Figure S32. Principal Component Analysis of Phage OES_C-2 illustrates distinct clustering patterns between putative virion proteins (PVP) and non-PVP proteins. The majority of proteins are densely clustered near the origin, suggesting shared physicochemical properties across most of the proteome. Notably, three PVP outliers are positioned at extreme locations, likely indicative of specialized structural components such as tail fiber proteins or receptor-binding proteins with unique sequence characteristics. Additionally, two non-PVP outliers are observed, which may represent proteins with atypical properties, potentially involved in specialized DNA metabolism, regulatory, or lytic functions.

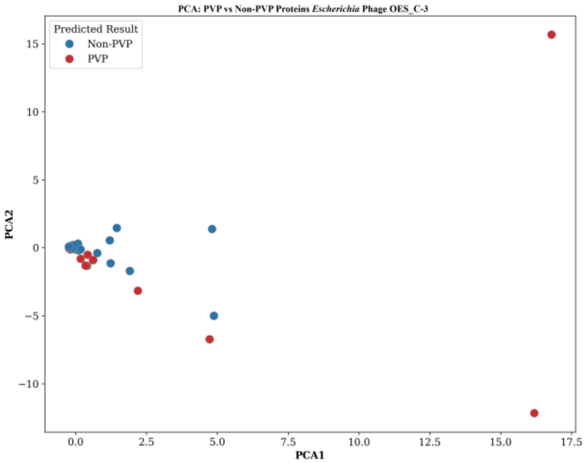

Figure S33. Principal Component Analysis of Phage OES_C-3 reveals clustering patterns closely resembling those of Phage_OES_C-1 and Phage_OES_C-2. This similarity underscores a conserved proteome organization across the OES phage series. The majority of proteins are densely clustered near the origin, suggesting shared physicochemical properties. Notably, three PVP outliers are located at extreme coordinates, likely indicative of specialized tail fiber proteins or receptor-binding components with distinct sequence characteristics. Additionally, two non-PVP outliers suggest proteins with specialized functions, potentially involved in DNA metabolism or regulatory processes.

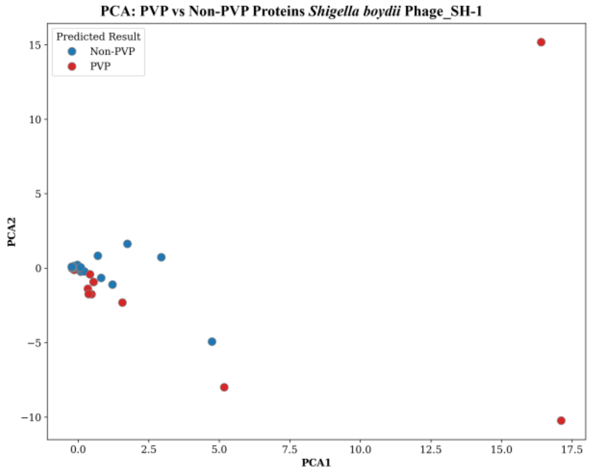

Figure S34. Principal Component Analysis of Phage_SH-1 illustrates distinct clustering patterns between putative virion proteins (PVP) and non-PVP proteins. The majority of proteins are densely clustered near the origin, indicative of shared physicochemical properties characteristic of core phage proteins. Notably, four PVP outliers are located at extreme coordinates, representing specialized structural components such as tail fiber proteins with unique receptor-binding domains. Additionally, one non-PVP outlier suggests a protein with specialized functions, potentially involved in DNA metabolism, recombination, or host lysis mechanisms.

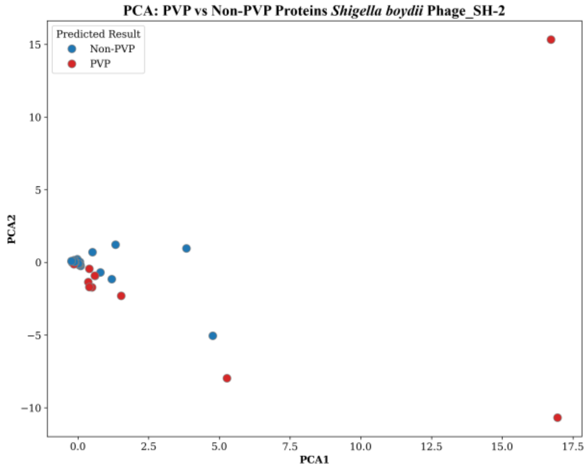

Figure S35. Principal Component Analysis of Phage_SH-2 demonstrates distinct clustering patterns between PVP and non-PVP proteins, with notable outlier distribution. The majority of proteins are densely clustered near the origin, indicative of shared physicochemical properties characteristic of core phage proteins. Notably, three PVP outliers are located at extreme coordinates, with two positioned at opposite poles, representing specialized structural components such as tail fiber proteins, baseplate components, or highly modified capsid decoration proteins with distinct receptor-binding specificities. Additionally, non-PVP outliers are present, with one notable protein suggesting specialized functions potentially involved in host cell wall degradation, anti-restriction systems, or DNA recombination pathways.

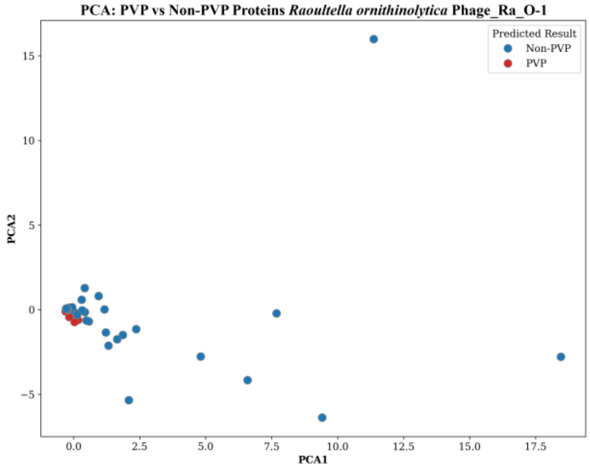

Figure S36. Principal Component Analysis of Phage_Ra_O-1 demonstrates a limited presence of PVP proteins, with a predominant distribution of non-PVP proteins. The majority of proteins are densely clustered near the origin, indicating shared physicochemical properties characteristic of core phage proteins. Notably, the four identified PVP proteins cluster tightly within the main group, suggesting conserved structural components with similar biochemical features. Several non-PVP outliers are positioned at extreme coordinates, representing proteins with specialized functions potentially involved in DNA replication, lysogeny-related mechanisms, or metabolic processes essential for phage propagation within the host.

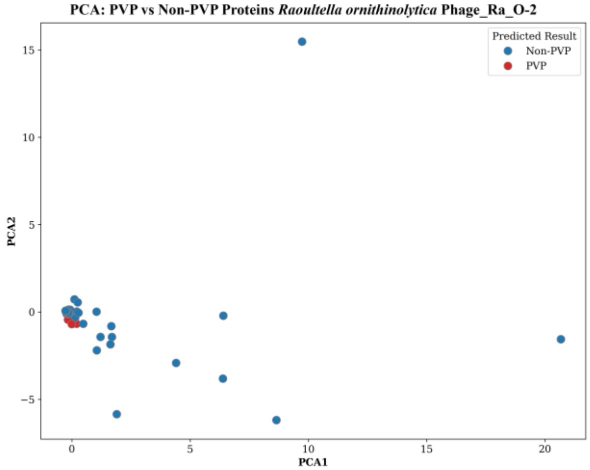

Figure S37. Principal Component Analysis of Phage_Ra_O-2 reveals clustering patterns analogous to those of the closely related Phage_Ra_O-1, characterized by limited PVP representation and substantial non-PVP protein diversity. The majority of proteins cluster densely near the origin, indicative of shared physicochemical properties typical of core phage proteins. The five identified PVP proteins exhibit tight clustering near the origin, suggesting conserved virion structural components, such as major capsid and portal proteins. Several non-PVP outliers are distributed across the plot, with one extreme outlier representing proteins with specialized functions potentially involved in DNA packaging, transcriptional regulation, or auxiliary metabolic genes that enhance phage fitness during infection.

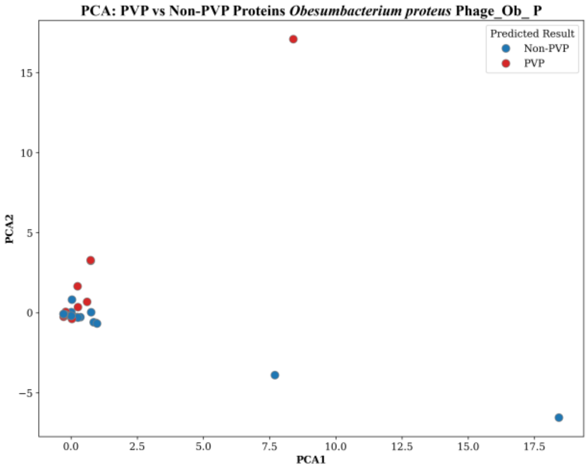

Figure S38. Principal Component Analysis of Phage_Ob_P reveals distinct clustering patterns between putative virion proteins (PVPs) and non-PVP proteins. The majority of proteins cluster densely near the origin, indicative of shared physicochemical properties characteristic of core phage proteins. Notably, one prominent PVP outlier occupies extreme coordinates, potentially representing a specialized structural component, such as a tail fiber protein with unique receptor-binding domains or a highly decorated capsid protein. Additionally, several non-PVP outliers are dispersed across the plot, with one particularly distant point suggesting proteins with specialized functions potentially involved in DNA metabolism, recombination, or host lysis mechanisms.

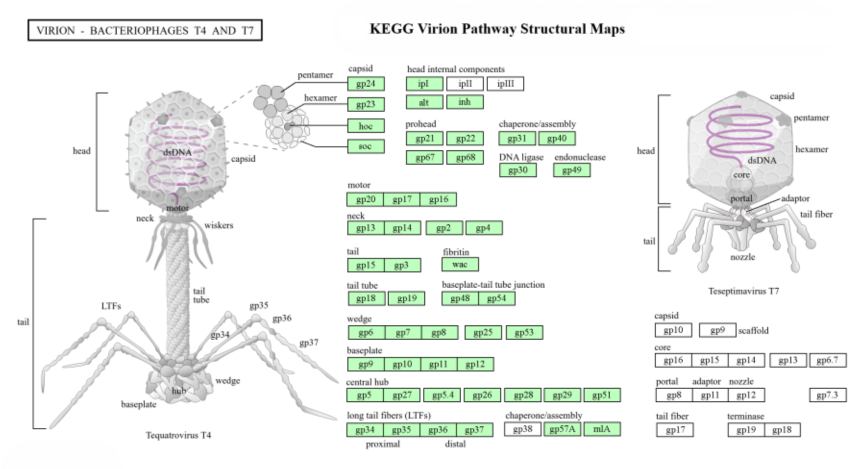

Figure S39. A comparative diagram depicts the protein structural pathway for Phages OES_C-3 and SH-1, with bacteriophage T4 serving as a reference model. Both phages exhibit an identical structural organization, with genes hierarchically arranged from head to tail as follows: capsid assembly (gp24 pentamer, gp23 hexamer, hoc, soc), head internal components (ipI, alt, inh), prohead formation (gp21, gp22, gp67, gp68), chaperone and assembly factors (gp31, gp40), enzymatic components (gp30 DNA ligase, gp49 endonuclease), DNA packaging motor (gp20, gp17, gp16), neck structure (gp13, gp14, gp2, gp4), tail sheath (gp15, gp3), fibritin (wac), tail tube (gp18, gp19), baseplate-tail tube junction (gp48, gp54), wedge proteins (gp6, gp7, gp8, gp25, gp53), baseplate assembly (gp9, gp10, gp11, gp12), central hub (gp5, gp27, gp5.4, gp26, gp28, gp29, gp51), and long tail fiber assembly with proximal and distal fibers (gp34, gp35, gp36, gp37), facilitated by chaperone factors (gp57A, mlA).

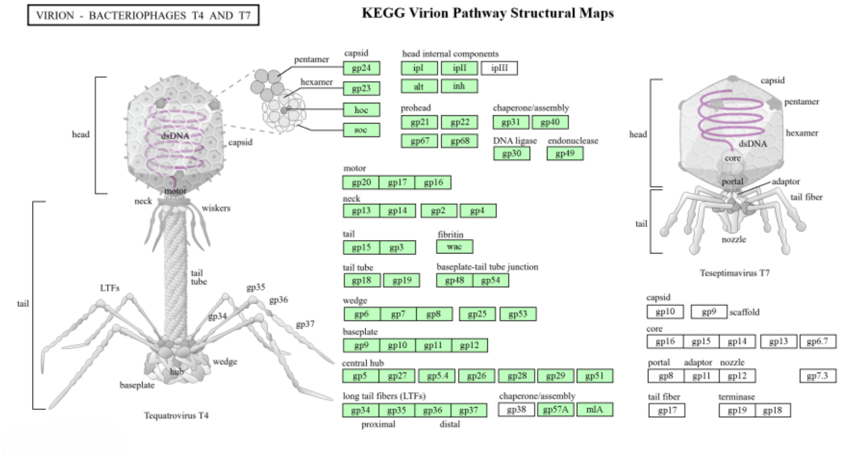

Figure S40. A comparative diagram presents the protein structural pathway for Phage_SH-2 using bacteriophage T4 as reference model. The structural pathway for Phage_SH-2 is represented by the following genes organized hierarchically from head to tail: capsid (gp24 pentamer, gp23 hexamer, hoc, soc) head internal components (ipI, ipII; alt, inh) prohead (gp21, gp22, gp67, gp68) chaperone/assembly (gp31, gp40) DNA ligase/endonuclease (gp30, gp49) motor (gp20, gp17, gp16) neck (gp13, gp14, gp2, gp4) tail (gp15, gp3) fibritin (wac) tail tube (gp18, gp19) baseplate-tail tube junction (gp48, gp54) wedge (gp6, gp7, gp8, gp25, gp53) baseplate (gp9, gp10, gp11, gp12) central hub (gp5, gp27, gp5.4, gp26, gp28, gp29, gp51) long tail fibers (gp34, gp35, gp36, gp37, proximal and distal) chaperone/assembly (gp57A, mlA).

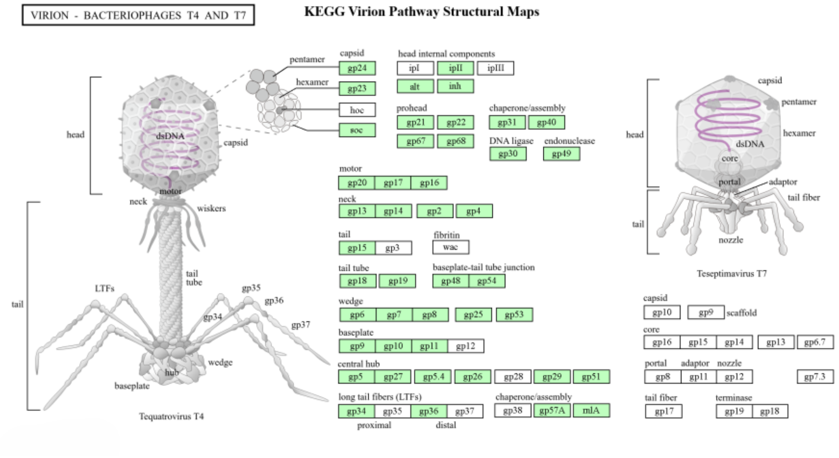

Figure S41. A comparative diagram illustrates the protein structural pathway for Phages Ra_O-1 and Ra_O-2, employing bacteriophage T4 as a reference model. Both phages exhibit a streamlined structural organization, with genes hierarchically arranged from head to tail as follows: capsid assembly (gp24 pentamer, gp23 hexamer, with Ra_O-2 additionally incorporating soc), head internal components (ipII, alt, inh), prohead formation and chaperone factors (gp21, gp22, gp67, gp68, gp31, gp40), enzymatic components (gp30 DNA ligase, gp49 endonuclease), DNA packaging motor (gp20, gp17, gp16), neck structure (gp13, gp14, gp2, gp4), tail sheath (gp15), tail tube (gp18, gp19), baseplate-tail tube junction (gp48, gp54), wedge proteins (gp6, gp7, gp8, gp25, gp53), baseplate assembly (gp9, gp10, gp11), central hub (gp5, gp27, gp5.4, gp26, gp29, gp51), and long tail fiber assembly (gp34, gp36) facilitated by chaperone factors (gp57A, mlA). This configuration represents a simplified two-component tail fiber system in comparison to canonical T4-like phages.
